# Impact of Physicochemical Parameters on the Diversity and Distribution of Microbial Communities Associated with Three South African Peatlands

**DOI:** 10.3390/microorganisms10112103

**Published:** 2022-10-23

**Authors:** Shandré S. L. Weels, Pamela J. Welz, Alaric Prins, Marilize Le Roes-Hill

**Affiliations:** 1 Applied Microbial and Health Biotechnology Institute, Cape Peninsula University of Technology, Bellville Campus, P.O. Box 1906, Bellville 7530, South Africa; 2 Department of Environmental and Occupational Studies, Cape Peninsula University of Technology, District Six Campus, P.O. Box 652, Cape Town 8000, South Africa

**Keywords:** wetland, peatlands, actinobacteria, basidiomycetes, ascomycetes, physicochemical parameters, South Africa

## Abstract

Peatlands are complex wetland-like ecosystems that harbor diverse microbial communities. In this study, the microbial communities (fungal and actinobacterial) associated with an unimpacted peatland (Vankervelsvlei; VV), an impacted peatland (Goukou River system; GK), and a developing peatland (Nuwejaars River system; NR) were determined through ITS and 16S rRNA metataxonomic analyses. Unidentified *Acidimicrobiales* dominated in GK and NR, unidentified *Intrasporangiaceae* and *Solirubobacterales* in NR, and *Corynebacterium*, *Propionibacterium*, and *Streptomyces* species in VV. The fungal phyla, *Ascomycota* and *Basidiomycota*, dominated all three sites, and harbored unique fungal taxa belonging to a wide range of fungal guilds. Physicochemical properties of the peat collected from the three sites were analyzed in association with microbial community structures in order to determine which parameters acted as the main drivers for microbial diversity. BEST analysis (linking microbial diversity patterns to environmental variables) showed that nitrogen (N), aluminum (Al), phosphorus (P), and potassium (K) were the most significant physicochemical drivers of actinobacterial community structure, while iron (Fe) and humification were the environmental parameters that affected the fungal communities the most. In conclusion, this study has provided some insight into the fungal and actinobacterial communities associated with three South African peatlands and the main environmental drivers that influence these communities.

## 1. Introduction

Globally, peatlands are widespread, covering 3% or 4.16 million km^2^ of terrestrial and freshwater land surface, of which an estimated 80% are found in the temperate-cold climates of the Northern Hemisphere [1]. The slow decay of plant material under sustained waterlogged conditions results in the formation of a brown-black organic-rich soil called peat [2]. This formation is mainly due to the breakdown of plant material by aerobic and anaerobic microorganisms. However, a point is reached where there is an imbalance in the influx of plant (organic) material and its breakdown/removal by microbial communities. This is mainly due to a lack of access to organic material, the presence of organic material with low or no degradative qualities, low temperatures, low pH, low nutrient levels, the level of inflowing water, and oxygen availability [3,4]. The type of organic compounds found in peatlands vary from site to site. Peat typically contains organic compounds containing nitrogen (N) and/or sulfur (S), as well as aromatic compounds such as tannins, lignin and lignin-like compounds, lipids, and proteins, with lignin-like compounds dominating (34–57% of the total) [5]. It is generally known that it is the action of microorganisms that controls the organic carbon (C) turnover, nutrient mineralization, and uptake in peatlands [6]. Various studies have shown that microbial populations are influenced by the environmental conditions associated with the peatlands, with the type of vegetation being one of the main driving forces [7,8]. It was initially thought that the decomposition of plant material was mostly due to the action of fungal strains, but recent studies have shown that bacteria may play a more active role than fungi and that the decomposition process is due to a consortium of microorganisms with complementary enzyme activities [6,9].

Peatlands provide valuable ecosystem services such as hydrological buffering and climate change mitigation through carbon storage [8]. Worldwide, peatlands are impacted by human activities such as peat mining, water abstraction, afforestation, grazing, agriculture, and erosion, as well as the building of roads, fences, and dams [8,10]. The restoration of these peatlands is a time-consuming process and can take decades for the peatland to reach a functional state similar to the conditions prior to the disturbance. Studies by Anderson et al. [11,12] showed that the success of restoration is not only dependent on re-establishing the vegetation (e.g., *Sphagnum* species), but that microbial communities involved in the biogeochemical cycling also need to be restored, a process that takes time. A study by Nwaishi et al. [13] summarized the various parameters that should be used to monitor the ‘health’ of a restored or constructed peatland. Among these, biodiversity and trophic interactions are considered to be functional characteristics that can be monitored either by examining the functional microbial diversity or the species richness for various taxa.

The occurrence of peatlands in South Africa is often considered a mystery. South Africa has a low average rainfall and a high evaporation rate (up to 200%) when compared to the areas in the Northern Hemisphere with a known abundance of sphagnum-associated peatlands [2,14]. Eleven peatland eco-regions have been identified in South Africa, and each eco-region is characterized by different dominant, well-documented vegetation types ranging from fynbos to mosses [10]. Even though extensive research has been performed to determine the microbial populations associated with peatlands, no study has been performed on peatlands found in South Africa. Therefore, the main aim of the work reported here was to determine the actinobacterial and fungal communities associated with three peatland sites in South Africa—an undisturbed peatland (Vankervelsvlei, VV), an impacted peatland (Goukou River system, GK), and a developing peatland (Nuwejaars River system, NR)—as well as the role of the physicochemical properties of the three environments in driving diversity and composition. The specific objectives included determining (1) the composition of the actinobacterial and fungal communities associated with each site, (2) the physicochemical properties of each site, and (3) the role of the physicochemical parameters as drivers of microbial community composition.

## 2. Materials and Methods

### 2.1. Sample Collection

Samples were obtained from three different wetland systems in the Western Cape province of South Africa: VV near Sedgefield (34.01167 S 22.90333 E), GK near Riversdale (34.02361 S 21.30333 E), and NR located on the Agulhas plain (34.74111 S 19.91222 E). The sample collection from VV is described in Strobel et al. [15], which focused on the geochemical properties, age, macrofossil composition, and micropaleontology properties of the samples. For GK and NR, triplicate samples were collected: topsoil (0 to 5 cm depth), designated as ‘top’; samples from 25 cm depth, designated as ‘middle’; and samples from 50 cm depth, designated as ‘bottom’. This sampling approach was repeated at 30 m intervals. In total, 27 samples were collected at GK and 18 from NR. For subsequent analyses, all triplicate samples for GK and NR were combined, resulting in 9 samples from GK, 6 from NR, and 12 from VV. All samples were kept cool during transport and were processed within two days of collection.

### 2.2. Determination of Physicochemical Properties

Peat samples were submitted to Bemlab (Somerset West, South Africa) for C, hydrogen (H), and N analysis by elemental analyzer and to the Central Analytical Facility at the Stellenbosch University for analysis of major elements by X-ray fluorescence (XRF). In order to determine the pH of the samples, 10 mL of 0.01 M CaCl_2_ was added to 10 g of peat. The peat–CaCl_2_ was stirred and allowed to settle for 1 h. The pH meter (pH700 meter and probe, Eutech Instruments, Singapore) was calibrated (according to the manufacturer’s instructions), and the pH of the peat samples measured and recorded (in triplicate). For the samples VV5 and VV10, litmus paper was used due to the small sample size.

For the determination of organic matter content, 20 g of peat was weighed out and dried for 24 h at 100 °C. After 24 h, the crucibles were placed in a desiccator for 30 min, allowing the samples to cool. The mass of the peat samples was recorded. The peat samples were placed in a furnace at 550 °C for 4 h. After 4 h, the crucibles were placed in a desiccator, allowing the sample to cool down, and the recorded mass and percentage of organic matter were calculated using Equation 1 [16].

Organic matter content % = [(dried weight at 100 °C − weight after ashing at 550 °C)/dried weight at 100 °C] × 100(1)

The method described by Blackford and Chambers [17] was used to determine peat humification. All physicochemical data are provided as Supplementary Material (Tables S1 and S2).

### 2.3. DNA Extraction and Library Preparation

Metagenomic DNA was extracted using the DNeasy® PowerSoil® kit (QIAGEN) with the following modification: 1 g (instead of 0.25 g) of the sample was used for the extraction. A control (no soil) was included during the processing to ensure that no contaminants were introduced during the isolation of metagenomic DNA. In order to determine actinobacterial diversity, the actinobacterial-specific 16S rRNA gene primer pair (Com2xf: 5′-AAACTCAAAGGAATTGACGG-3′; Ac1186r: 5′-CTTCCTCCGAGTTGACCC-3′) [18] was used, while fungal diversity was determined using the ITS1F (5′-CTTGGTCTTTAGAGGAAGTAA-3′) and ITS4Rv2 (5′-TCCTCCGCTTATTGATATGC-3′) primer pair [19,20]. Illumina adapters and barcodes were incorporated by the high-throughput sequencing service provider, Inqaba Biotechnical Laboratories (Pretoria, South Africa). Amplicon libraries were purified using the Agencourt Ampure® XP bead kit (Beckman Coulter, Brea, CA, USA) and sequenced on an Illumina MiSeq™ instrument. Raw reads were pre-processed and supplied as .fastq files (NCBI BioProject accession number: PRJNA805212).

### 2.4. Data Processing

#### 2.4.1. Metataxonomics

Metataxonomic analysis was performed using QIIME v1.9.1 [21] in a miniconda3 environment. The quality of the pre-processed reads (demultiplexed, barcodes, and indices removed) was determined using FastQC. Quality filtering and trimming of reads were executed prior to combining reads into a single sequence library. Open-reference OTU picking was performed using the EZBioCloud 16S v1.5 [22] and UNITE ITS 12_11 [23] databases for 16S rRNA gene and ITS gene assignments, respectively. All code and logs pertaining to the generation of the metataxonomic data as well as the taxa output file are supplied as Supplementary Information. Venn diagrams were generated to represent the number of unique and shared actinobacterial and fungal OTUs. The trophic modes of the fungal communities and shared fungal OTUs were determined by using FUNGuild [24]. The dataset could be divided into three trophic modes: pathotroph, symbiotroph, and saprotroph, and various combinations of these. Within these trophic modes, fungi were further divided into a total of 73 guilds (Supplementary Materials: Excel sheet otu_table.guilds_matched). Species richness and diversity were also determined using the phyloseq package [25] in RStudio (v 2022.07.0 build 548). Graphs were drawn in RStudio using ggplot v3.3.6. For both the fungal and bacterial communities, organisms predominantly driving the communities were identified using the labdsv package v 2.0-1 in RStudio. The indicator value of a species reflects its fidelity and relative average abundance. The R scripts used are available in the Supplementary Materials.

#### 2.4.2. Community Analyses

Statistical analyses were performed using Primer 7^®^ software (Quest Research Limited, Auckland, New Zealand) according to the developer’s instructions [26,27]. Analysis of similarity (ANOSIM) was based on Spearman rank similarity. Multivariate analyses on square root and fourth root transformed data were performed on the actinobacterial and fungal NGS data and the physicochemical data, respectively. For the microbial communities, non-metric multidimensional scaling (nMDS) plots and cluster plots (based on group average linkage) were constructed from Bray–Curtis similarity matrices, while for the physicochemical data, principal component analysis (PCA) plots and cluster plots (based on group average linkage) were constructed from similarity matrices based on Euclidean distances. To assess the effect of physicochemical parameters on the microbial community structures, the most important drivers were determined using BEST analyses. The physicochemical dataset was reduced to the maximum number of parameters permissible for BEST analyses. Firstly, a co-linearity check was performed on 4th root transformed and normalized physicochemical data. It was found that the (Spearman) correlation co-efficient was >0.95 for S + N, C + N, loss on ignition (LOI) + N, and LOI + C, allowing one parameter to be chosen as a proxy for the group of parameters. A separate BEST analysis using only these parameters was run separately for actinobacteria and fungi, and the highest respective R values were found to be for N (0.420, *p* ≤ 0.05) and S (R = 0.295, *p* ≤ 0.05); therefore, these were included as the proxy values for actinobacteria and fungi, respectively. Secondly, separate BEST analyses were run for actinobacteria and fungi using the parameters determined using XRF. The highest significant (*p* ≤ 0.05) global R values of 0.676 and 0.596 were obtained from combinations of aluminum (Al), calcium (Ca), phosphorus (P), and titanium (Ti) (actinobacteria), and Al, Ca, potassium (K), P, and Ti (fungi), respectively. The parameters that were included in the final BEST analyses were selected on the highest single R values obtained. The final physicochemical dataset for actinobacterial BEST analysis was N (as proxy for N, S, C, LOI), Al, Ca, P, Ti, K, silica (Si), iron (Fe), magnesium (Mg), humification, and pH. The final physicochemical dataset for fungal BEST analysis was S (as proxy for N, S, C, LOI), Al, Ca, K, P, Ti, Si, Mg, Fe, humification, and pH.

To determine the most important drivers (and the concentrations thereof) for the selection of the microbial community structures, constrained cluster analyses (LINKTREE) were performed using Bray–Curtis similarity matrices (microbial) and the physicochemical datasets determined using BEST.

## 3. Results

### 3.1. Actinobacterial and Fungal Community Structures at Different Sites and Depths

**Figure 1:**
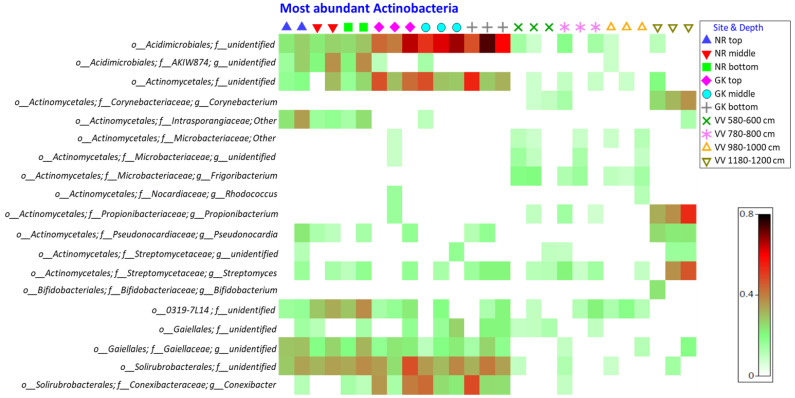
Shade plot of square root transformed relative abundance of the most abundant actinobacterial operational taxonomic units in the peat samples collected from the three study sites. NR = Nuwejaars River system (developing peatland); GK = Goukou River system (impacted peatland); VV = Vankervelsvlei (undisturbed peatland). Top: samples from 0 to 5 cm depth; middle: samples from 25 cm depth; bottom: samples from 50 cm depth. o = order; f = family; g = genus.

The actinobacterial orders *Acidimicrobiales, Actinomycetales, Gaiellales*, and *Solirubrobacterales* were present in the highest relative abundances (RA) at all three sampling sites from 0 to 50 cm depth (Figure 1), further supported by the indicator species analysis where these taxa occur in the highest frequencies (Table 1). An increase in RA for *Acidimicrobiales, Actinomycetales, Gaiellales*, and *Solirubrobacterales* was observed with an increase in sampling depth. Less abundant actinobacteria included *Micrococcales, Euzebayles, Nitriliryptorales*, and *Rubrobacterales*, only in the NR samples, while *Bifidobacteriales* was only observed in one of the deepest VV samples. *Acidimicrobiales* was observed in high abundance, particularly in the GK samples (up to 29.05%).

**Figure 2:**
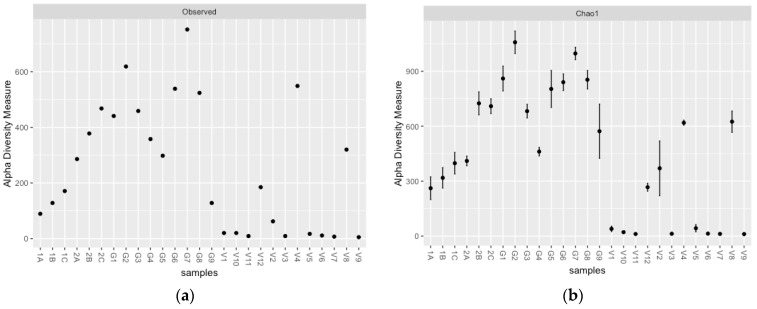

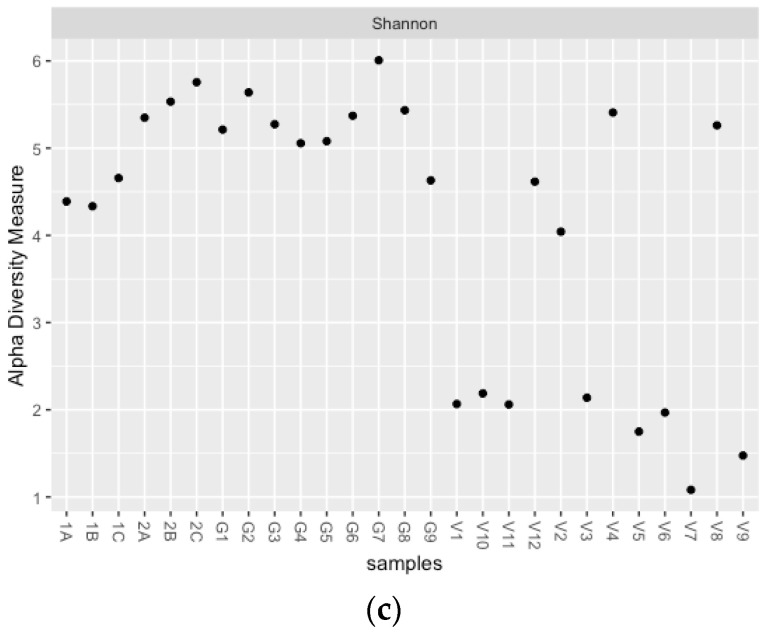
Actinobacterial species richness observed (**a**) and predicted (**b**) for the NR, GK, and VV samples, as well as the actinobacterial diversity (Shannon index; (**c**)). NR = Nuwejaars River system (1A–2C; developing peatland); GK = Goukou River system (G1–G9; impacted peatland); VV = Vankervelsvlei (V1–V12; undisturbed peatland).

The observed species richness and the predicted species richness (Figure 2a,b) indicated a higher overall species richness for GK compared to NR and VV, with VV exhibiting the lowest species richness. Greater actinobacterial diversity (Shannon index > 4) was also observed for NR and GK compared to VV, except for the deepest VV samples (Figure 2c).

**Figure 3:**
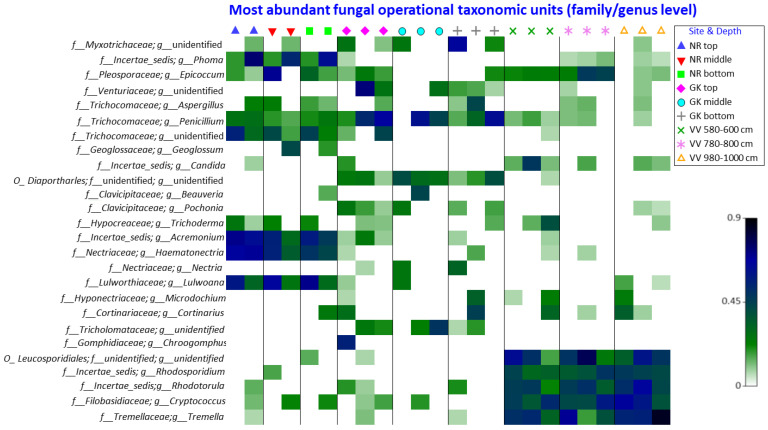
Shade plot of square root transformed relative abundance of the most abundant fungal operational taxonomic units in the peat samples. NR = Nuwejaars River system (developing peatland); GK = Goukou River system (impacted peatland); VV = Vankervelsvlei (undisturbed peatland); Top: samples from 0 to 5 cm depth; middle: samples from 25 cm depth; bottom: samples from 50 cm depth.

**Figure 4:**
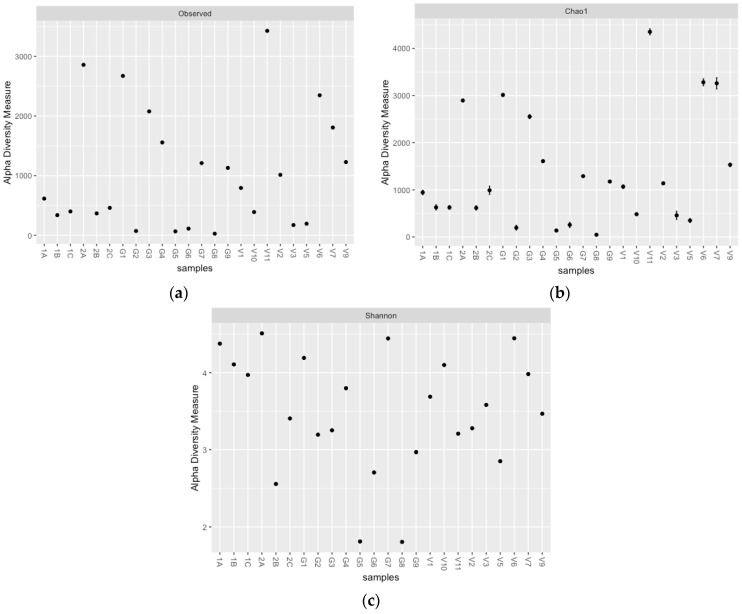
Fungal species richness observed (**a**) and predicted (**b**) for the NR, GK, and VV samples, as well as the fungal diversity (Shannon index; (**c**). NR = Nuwejaars River system (1A-2C; developing peatland); GK = Goukou River system (G1–G9; impacted peatland); VV = Vankervelsvlei (V1–V12; undisturbed peatland).

Five different fungal phyla (*Ascomycota, Basidiomycota, Chytridiomycota, Glomeromycota*, and *Zygomycota*) were detected in this study, representing 20 fungal classes, 53 orders, and 248 genera. Of the phyla detected, the *Ascomycota* and *Basidiomycota* dominated all three environments (Figure 3), with *Basidiomycota* being the dominant phylum in VV, *Ascomycota* in NR, and unidentified fungi for GK. Of the *Basidiomycota*, *Agaricomycetes* were the most dominant for NR and GK, while *Microbotyomycetes* and *Trellomycetes* were the most dominant fungal classes detected. The *Ascomycota*, *Dothideomycetes*, and *Sordariomycetes* were found in all sites, with the latter class being the most dominant for NR. *Eurotiomycetes* was detected mostly in GK and NR samples and was the most dominant class for GK, followed by *Dothideomycetes*. For all three sampling sites, *Ascomycota* abundance decreased with depth, evidenced by the decrease in fungal diversity (Figure 4c). *Ascomycota* are also observed as the overall representative taxa per the indicator species analysis (Table 2). *Zygomycota* was only detected in the NR and GK samples. Shade plots of the relative abundance of fungal phyla, actinobacterial orders, and the fungal classes *Ascomycota* and *Basidiomycota* can be seen in Supplementary Figures S1 and S2.

For the fungal communities, the observed (Figure 4a) and predicted (Figure 4b) species richness decreased with an increase in sampling depth. In terms of diversity, the fungal communities were relatively even across the board and higher in diversity, bar a few middle and deep samples (Figure 4c).

**Figure 5:**
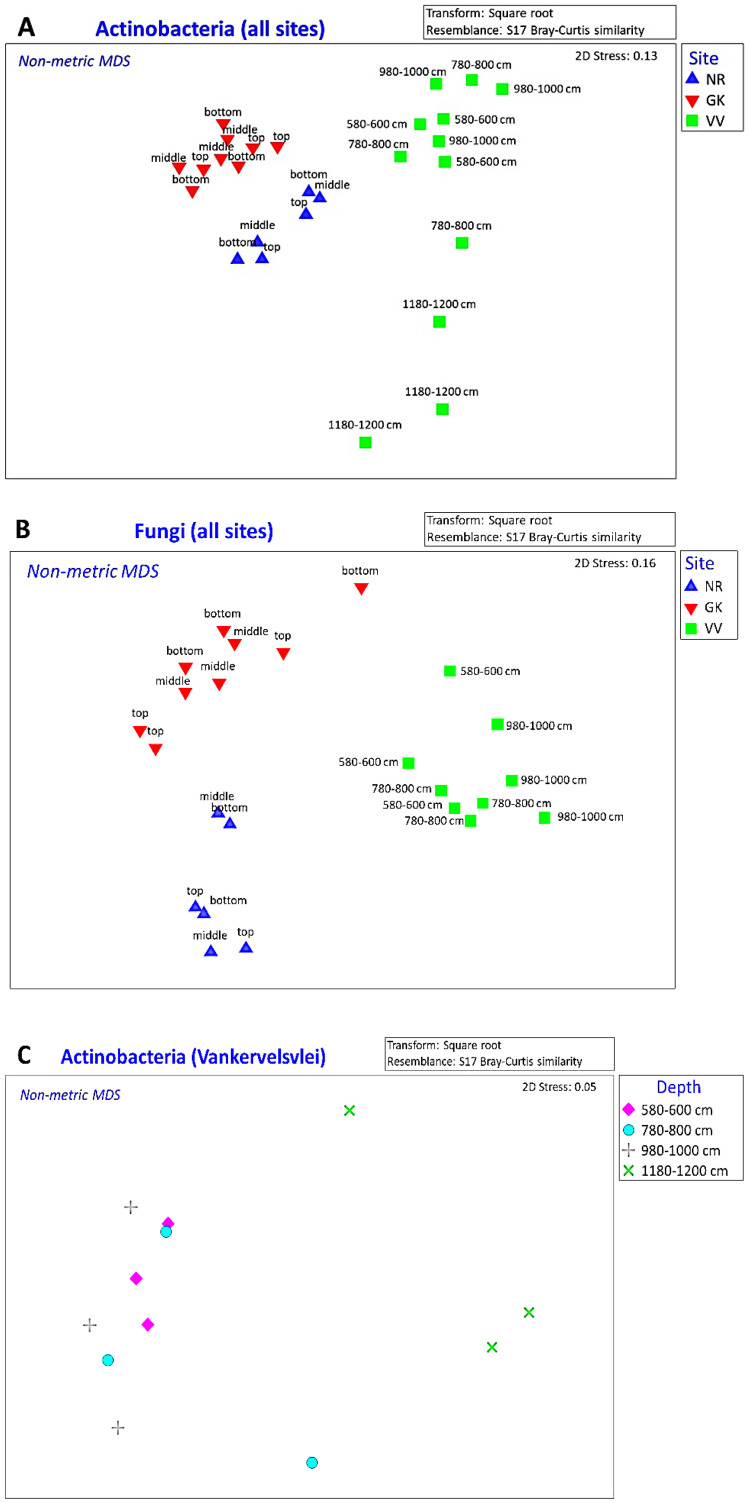
Non-metric multidimensional scaling plot showing similarities of the actinobacterial (**A**) and fungal (**B**) community structure from all the sites at different depths, and the actinobacterial community structure (**C**) from Vankervelsvlei at different depths. ‘Top’ = 0 to 5 cm depth, ‘middle’ = 25 cm depth, ‘bottom’ = 50 cm depth. NR = Nuwejaars River (developing peatland); GK = Goukou River (impacted peatland); VV = Vankervelsvlei (undisturbed peatland).

In this study, there were highly significant differences in the overall actinobacterial and fungal community compositions from the three peatlands, evidenced by ANOSIM (Table 3) and visualized using nMDS (Figure 5A,B) and cluster plots (Supplementary Figures S3 and S4). However, there were no significant differences in either the actinobacterial or fungal community compositions at different depths at NR or GK, or at depths <1180 cm at VV (ANOSIM *p* > 0.05).

Due to inherent differences in the geography of the sites, the depths of the peat layers, and the availability of more sophisticated sampling equipment at VV, deeper samples were taken at VV than at the other sites. The maximum depth of samples from GK and NR was 50 cm, while samples were taken up to 2000 cm at VV. The actinobacterial communities at depths > 1180 cm at VV were significantly different from those at depths ≤ 1000 cm (Table 4, Figure 5C). It is possible that the fungal communities at VV were also different at ≥1180 cm, but as part of the original study rationale, samples were not processed using ITS primers at this depth, as according to the literature, fungi are not expected to play functional roles in deeper layers of peatlands [28]. The comparatively low organic matter concentrations and clay-like consistency of the deep (1180–1200 cm) samples in comparison to the peaty appearance and texture of the other samples from VV strongly suggested that the bed of the peatland had been reached with the sampling equipment 1180 cm below the surface at that particular sampling site at VV.

**Figure 6:**
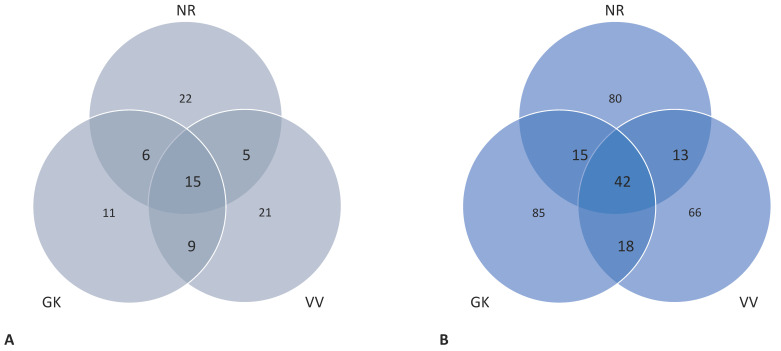
Venn diagrams indicating the number of unique and shared OTUs detected for the actinobacterial communities (**A**) and the fungal communities (**B**); NR = Nuwejaars River system (developing peatland); GK = Goukou River system (impacted peatland); VV = Vankervelsvlei (undisturbed peatland).

An analysis of the distribution of unique and shared OTUs showed that the three sites only shared 15 actinobacterial OTUs but 42 fungal OTUs (Figure 6). Actinobacterial orders represented among the shared OTUs include *Acidimicrobiales* (families: unidentified and AKIW874), *Actinomycetales* (families: unidentified, *Intrasporangiaceae*, *Nocardioidaceae*, *Pseudonocardiaceae*, and *Steptomycetaceae*), 0319-7L14 (families: unidentified), *Gaiellales* (family: *Gaiellaceae*), and *Solirubrobacterales* (families: unidentified and *Conexibacteraceae*). Interestingly, there were far more fungal OTUs in common among the three sampling sites than there were shared actinobacterial OTUs. The *Ascomycota* represented the majority of the shared fungal OTUs (29/42), followed by the *Basidiomycota* (11/42), and two groups where assignment could not be made to the phylum level (Supplementary Materials). An analysis by FUNGuild made it possible to gain insights into the potential roles of the shared fungal taxa (Table 5). Not all of the taxa could be assigned with a trophic mode—only 26 of the 42 shared fungal OTUs could be assigned. Dominant fungal taxa associated with the GK, NR, and VV samples could be assigned to specific fungal guilds, which include pathogenic, parasitic, saprotrophic, and symbiotic fungal taxa (Table 6).

### 3.2. Physicochemical Profiles at Different Peatland Sites and Depths

**Figure 7:**
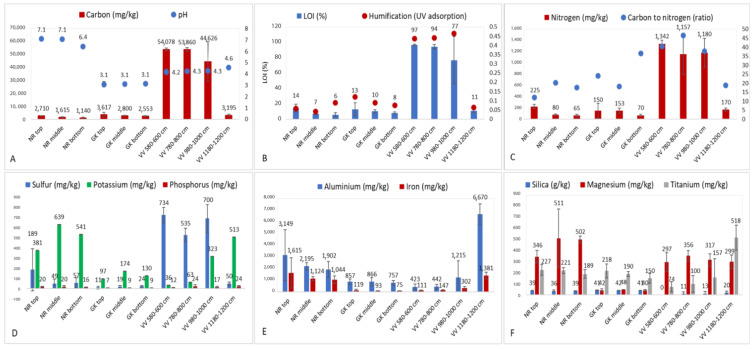
Measured parameters determined by BEST analyses to be the most important drivers of actinobacterial and/or fungal community structure (**A**–**F**) and pH (**A**) in samples taken from the study sites at different depths. LOI: loss on ignition. NR = Nuwejaars River system (developing peatland); GK = Goukou River system (impacted peatland); VV = Vankervelsvlei (undisturbed peatland). Top: samples from 0 to 5 cm depth; middle: samples from 25 cm depth; bottom: samples from 50 cm depth.

In this study, the pH values (Figure 7A) were significantly higher in the NR samples (*p* ≥ 0.5, ANOVA) compared to the low pH levels of GK and VV. With the exception of the deep samples (≥1180 cm), ostensibly from the peatland bed, the C concentrations (Figure 7A), LOI, and humification values (Figure 7B) were significantly higher in the VV samples than in the samples from the other sites. Pearson correlations between C and LOI (0.999), C and humification (0.841), and LOI and humification (0.847) were highly significant. In terms of the other macronutrients, the N and S concentrations were significantly higher in the VV samples (<1180 cm), and despite the high C concentrations, the C:N ratios were also significantly higher in these samples (Figure 7C,D). The K and P concentrations were significantly lower in the samples from GK than in samples from the other sites (Figure 7D). For the other important selective cations analyzed, (i) the Al and Fe concentrations were significantly lower in samples from GK and the <1180 cm samples from VV (Figure 7E), (ii) the Si concentrations were significantly lower in the VV samples, (iii) the Mg concentrations were significantly lower in the GK samples, and (iv) the Ti concentrations were highest in the deep VV (>1180 cm) samples (Figure 7F).

**Figure 8:**
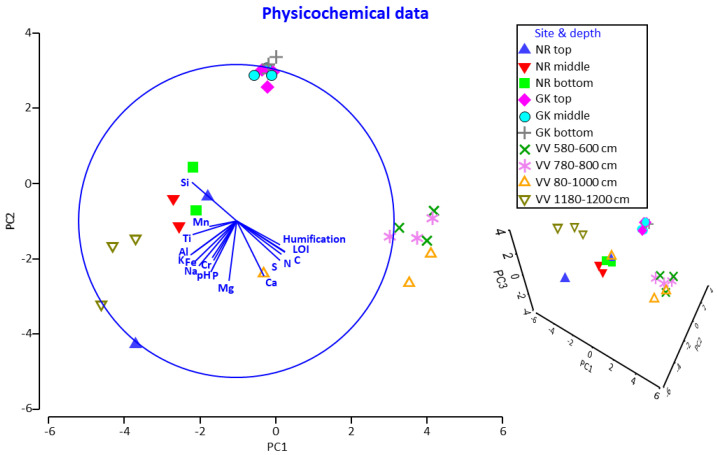
Two-dimensional and three-dimensional (insert bottom right) principal component analysis plots of normalized physicochemical data from the peat samples; 47.1%, 32.0% and 9.3% (cumulative 88.4%) of the variations are explained by PC1, PC2, and PC3, respectively. NR = Nuwejaars River system (developing peatland); GK = Goukou River system (impacted peatland); VV = Vankervelsvlei (undisturbed peatland). Top: samples from 0 to 5 cm depth; middle: samples from 25 cm depth; bottom: samples from 50 cm depth.

The PCA chart of the physicochemical profiles from the different sites and depths (Figure 8) showed: (i) close clustering of all the data points representing the samples from GK, (ii) close clustering of the data points representing the <1180 cm samples from VV, (iii) close clustering of the data points representing the deep (1180–1200 cm) samples from VV, (iv) large spatial differences between the data points representing the <1180 cm and 1180–1200 cm samples from VV, and (v) loose clustering of data points representing samples from GK, but no notable overlaps with data points representing samples from other sites. In terms of the overall physicochemical profiles for each site and depth, no significant differences (*p* > 0.05) were found for the factor ‘depth’ in samples from NR or GK. Overall, the differences in the individual physicochemical parameters (Figure 7) and physicochemical profiles in samples from the three sites (Figure 8) closely mirrored the results obtained from multivariate statistical analyses of the actinobacterial and fungal microbial community compositions. This clearly signified that the selected abiotic parameters played a pivotal role in driving actinobacterial and fungal microbial community selection.

### 3.3. Physicochemical Parameters as Drivers for Actinobacterial and Fungal Community Compositions

**Figure 9:**
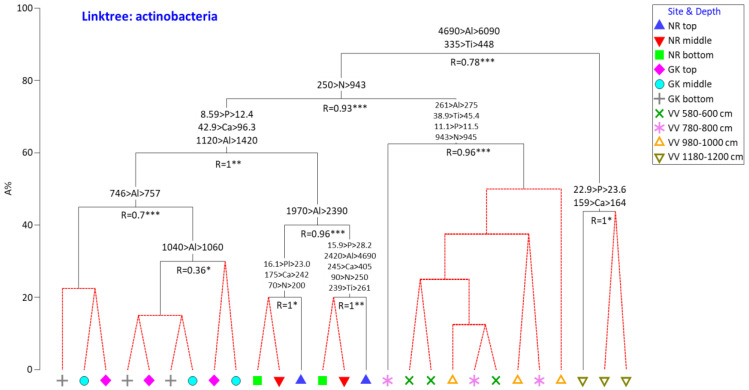
LINKTREE showing concentrations of the most important environmental parameters identified using BEST analysis selecting the actinobacterial community structure in the peat samples. All concentrations are in mg/L. N is a proxy for C, S, and LOI (loss on ignition). NR = Nuwejaars River system (developing peatland); GK = Goukou River system (impacted peatland); VV = Vankervelsvlei (undisturbed peatland). Top: samples from 0 to 5 cm depth; middle: samples from 25 cm depth; bottom: samples from 50 cm depth. Levels of significance: * 0.05 > *p* ≥ 0.01 ** 0.01 > *p* ≥ 0.005 *** 0.005 < *p*.

According to the LINKTREE analyses, pH was not a dominant distinguishing driver of either actinobacterial or fungal community structures despite being notably higher in the NR than in GK or VV samples. In terms of actinobacteria (Figure 9), higher Al (>6090 mg/g) and Ti (>448 mg/g) were the primary parameters that differentiated the community compositions in the deep (1180–1200 cm) VV samples from those in the rest of the samples. High N concentrations (>943 mg/g) and by proxy higher S, C, and LOI were the primary selective drivers distinguishing the remaining VV actinobacterial communities from those in the GK and NR samples. Higher P, K, and Al distinguished the actinobacterial communities in the NR samples from those in the GK samples.

**Figure 10:**
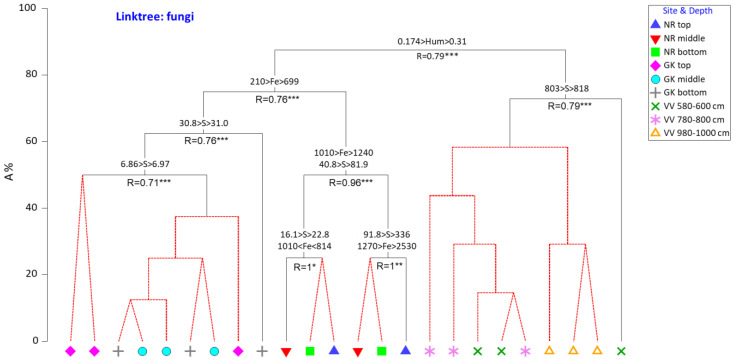
LINKTREE showing concentrations of the most important environmental parameters identified using BEST analysis selecting the fungal community structure in the peat samples. All concentrations are in mg/L except humification (Hum). S is a proxy for N, C, and LOI (loss on ignition). NR = Nuwejaars River system (developing peatland); GK = Goukou River system (impacted peatland); VV = Vankervelsvlei (undisturbed peatland). Top: samples from 0 to 5 cm depth; middle: samples from 25 cm depth; bottom: samples from 50 cm depth. Levels of significance: * 0.05 > *p* ≥ 0.01 ** 0.01 > *p* ≥ 0.005 *** 0.005 < *p*.

In terms of fungi (Figure 10), the degree of humification was the primary driver distinguishing the VV communities (>0.31 ABS) from those from GK and NR. Higher Fe concentrations (>699 mg/g) distinguished the fungal communities in the NR samples from those in the GK samples.

## 4. Discussion

Microorganisms are the key drivers of nutrient cycling and soil organic matter decomposition in peat ecosystems [4]. Fungi are often considered to be the primary decomposers and dominant microorganisms found in peat, and their ability to sequester C through the degradation of recalcitrant materials and their symbiotic relationship with plants make them essential to the maintenance of ecosystem equilibrium [3,4]. However, various studies have shown that actinobacteria also play a key role in mineralization processes and produce extracellular enzymes with comparable activities to those produced by fungi [29]. The ability of actinobacteria to degrade complex plant polymers such as cellulose, hemicellulose, and lignin enable them to survive in environments with low C availability [29]. Due to inherent climatic and physicochemical differences, it is expected that peat microbial communities will differ from site to site; it has also been shown that peatland depth may play a pivotal role in microbial community selection due to physicochemical differences such as the concentration and quality of organic matter, elemental composition, and moisture content of the peat [28,30,31]. Kotiaho et al. [32] also showed that the water table level and vegetation could influence peat microbial communities due to the effect these two factors have on oxygen availability and the quality of the organic substrates available.

In this study, three peatland sites were selected in order to gain insight into the differences in actinobacterial and fungal communities for an unimpacted peatland (VV), an impacted peatland (GK), and a developing peatland (NR). Actinobacteria were detected at all depths of the sites studied. In a study by Aksenov et al. [31] which focused on a permafrost peatland in Western Siberia, they found that the actinobacterial diversity decreased with depth, but found an increase in diversity at 90–100 cm, followed by a decrease at 100–110 cm. A similar phenomenon was observed for the VV samples in this study, and in both studies, actinobacteria were present over the full depth of the peat core taken. The authors concluded that it is most probably due to the ability of actinobacteria to utilize a broad range of substrates and their ease of adaptation to low temperatures [31]. The majority of soil actinobacteria are aerobic, but are tolerant of anaerobic conditions, with viable actinobacteria having been reported to be isolated from anaerobic peat samples further substantiating the detection of actinobacteria at a depth > 1000 cm [32,33].

Five different actinobacterial orders dominated the NR site (0319-7L14, *Acidimicrobiales* (AKIW874), *Actinomycetales* (*Intrasporangiaceae*), *Gaiellales* (*Gaiellaceae*), and *Solirurobacteriaceae*), one order dominated the GK site (*Acidimicrobiales*), and one order dominated the VV site (*Actinomycetales* (*Corynebacteriaceae*, *Propionibacteriaceae*, *Streptomycetaceae*)). These results are further corroborated through indicator species analysis, with 0319-7L14 and *Gaiellales* dominating in frequency (*p* < 0.05) in the NR samples, *Acidimicrobiales* occurring in the highest frequency in the GK samples, and *Corynebacterium* and *Propionibacterium* dominating in the VV samples (*p* < 0.05). In a study by Jenkins et al. [34], it was shown that actinobacterial genera, *Arthrobacter* and *Micrococcus*, were more prevalent in organic-rich environments, while as seen in the study reported here, the genera *Streptomyces* and *Acidimicrobium* are more prevalent in acidic soils, with the dominance of *Acidimicrobiales* in the GK samples likely being a result of the low pH of the peatland (pH 2.93–3.31). The presence of *Propionibacterium* in peat was also reported in a study by Oloo et al. [35], where they proposed that members of this genus can degrade carbohydrates and polymeric compounds, essentially contributing towards C turnover in peatlands. Genera belonging to the shared *Actinomycetales* families detected are well known to produce hydrolytic enzymes involved in the degradation of both plant and animal polymers, as well as the production of oxidative enzymes that play a role in the decomposition of lignocellulose, releasing dissolved organic carbon in the peat environment [9]. Very little is known about members of the *Acidimicrobiales* AKIW874 family, which has been proposed as an indicator taxon for watering events in a semiarid grassland [36]. It has been associated with increased titanium levels [37], the presence of a drilosphere (soil impacted by earthworm activity) [38], and excessive mineral fertilizer use [39]. Similarly, there are few reports on the actinobacterial order 0319-7L14. In a study by Pershina et al. [40], a core microbiome of different soil types was proposed. The authors analyzed six different types of soil found in Russia, Crimea, and Kazakhstan. From the 24 samples analyzed, a set of 47 taxa were identified as being core members. These taxa are proposed to be able to adapt to a broad range of soil environments, and include the actinobacteria AKIW874, 0319-7L14, *Micrococcaceae*, EB1017, *Agromyces*, *Mycobacterium*, *Kribbella*, *Streptomyces*, *Micrococcales*, *Rubrobacter, Gaiellaceae*, and *Solirubrobacterales*. In a similar study, Zhang et al. [41] also found that members of the genera *Gaiella, Solirubrobacter, Nocardioides, Mycobacterium*, and *Pseudonocardia* are core members of five different soil ecosystems collected from the Heihe River Basin, China. From their bacterial community studies, Shange et al. [42] also came to the same conclusion that members of *Solirubrobacterales* (similar to members of *Actinomycetales*) seem to be highly adaptive and have the ability to colonize different soil environments.

The occurrence of the five fungal phyla detected in this study corresponds to previous studies that focused on peat soil [3,43,44,45]. Members of the *Zygomycota* are not often reported in peatland studies and were only detected for the NR and GK sites. This is not surprising, since members of the *Zygomycota* are not capable of utilizing cellulose and sucrose degradation products as C sources, but rather use C substrates of animal and fungal origin (e.g., fungal hyphae) [45]. Based on the indicator species analyses, members of the phylum *Ascomycota* occurred in the highest frequency in all three sites, further corroborating the low levels of *Zygomycota* detected. The fact that *Mortierella* species (*Zygomycota*) can degrade chitin, an essential component of fungal hyphae, means that this phylum most probably also plays a key role in C cycling in peatlands, but is often overlooked [45,46]. A wide range of shared fungal taxa were detected, including pathogenic (e.g., *Sordariomycetes, Haematonectria*; *Tremellomycetes*, *Cryptococcus*), symbiotic (e.g., *Agaricomycetes*, *Cortinarius*; *Dothideomycetes*, *Pleospora*), and saprotrophic (e.g., *Eurotiomycetes, Penicillium*) fungal taxa. Pathogenic and parasitic fungi are generally poor competitors when it comes to accessing C from organic matter and utilize simple sugars and other compounds from their hosts, while saprotrophs have the ability to degrade various polymers (cellulose, hemicellulose, and pectin) and recalcitrant compounds (lignin, tannins, humic acids) [46]. Endophytes, epiphytes, and ectomycorrhizal fungi mostly have mutualistic associations with plants, ensuring plant health and fungal survival. Upon the aging and death of the plant, these fungi could become pathogenic, producing enzymes involved in the degradation of plant material [47]. From a summary of all the fungal guilds predicted for all three sites, it was clear that most of the OTUs could be assigned as undefined saprotrophs (59/253; generalists), followed by plant pathogens (36/253), lichenized (22/253), in a symbiotic relationship with algae [48], and ectomycorrhizal (13/253), which are found in association with roots or found in the immediate root environment/rhizosphere [46].

Based on the results obtained in this study, the NR site (if not impacted by human activity) could potentially develop into a rich fen fed by groundwater and some rainfall. Unlike the more mineral-poor GK and VV peatlands, the area surrounding NR contains high concentrations of mineral deposits [49,50]. In addition, while the NR peatland is developing, it has been estimated that the deeper deposits at GK and VV are >6000 years old [49,50,51]. The GK river catchment area and associated peatlands are dominated by palmiet vegetation, and the peatlands are degraded due to agricultural activities and agricultural run-off [50]. In contrast, the VV site is a depression which is not anthropogenically impacted, is fed by groundwater as well as rainfall, and is dominated by a floating mass of sphagnum moss [51].

When statistical analyses were performed, it was found that although the samples from NR and GK were only extracted to a depth of 50 cm, the general lack of significant differences in the actinobacterial and fungal community compositions with depth at all three sites was not expected. In a study on Malaysian forest peatlands, for example, both depth (0–20 cm versus 30–50 cm) and site were both found to be significant drivers of prokaryotic community composition [9]. Similarly, Tian et al. [52] found that depth played a significant role in the actinobacterial and fungal community composition and bacterial and archaeal community composition in peatlands. However, these peatlands were located at high altitudes (>3400 m above sea level) and therefore experienced much lower temperatures [52,53] than the South African coastal peatlands.

Although many studies have focused on peatland vegetation, it has recently been suggested that abiotic factors and not vegetation types are the primary drivers of microbial community composition in peatlands [28]. Various environmental factors can serve as drivers for microbial communities: pH, temperature, soil moisture, organic matter content, nutrient availability, and biotic interactions (e.g., effect of plants on microorganisms, effect of bacteria on fungi, and vice versa) [4,9]. Peatland bogs are typically acidic, while the pH of minerotrophic fens can range from acidic to alkaline [54]. In this study, the pH values were significantly higher in the NR samples (*p* ≥ 0.5, ANOVA), substantiating the designation of this peatland as a developing rich fen. Furthermore, Pearson correlations between C and LOI (0.999), C and humification (0.841), and LOI and humification (0.847) were highly significant, indicating the abundance of humified organic matter at the undisturbed VV site in comparison to the low abundance at the minerotrophic NR and degraded GK. Lower concentrations of organic matter are expected in minerotrophic fens such as NR [55,56] and degraded peatlands such as GK [57]. The results obtained from the analysis of the macronutrients present in the three sites substantiate those found by Tipping et al. [58], who also reported a correlation between high N, P, and S in organic-rich peat.

Analysis of the effect of physicochemical parameters on actinobacterial and fungal community distribution showed that pH is not the main driver for selection. However, the pH of the environment could play an indirect role; for example, in acidic soils, there is an increased amount of soluble ionic metal(loid)s [59]. In contrast, it was found that Al, K, and P were the main drivers for actinobacterial community selection. Actinobacteria have developed various mechanisms to minimize the potential toxic effects of metal(loid)s found in their immediate environment [60]. In the case of Al, selected actinobacterial genera produce siderophores that can bind and remove the Al from the environment. Aluminum is one of several heavy metals that stimulate siderophore production [61]. The Al^3+^–siderophore complex thereby alleviates Al toxicity. One of the most abundant actinobacterial taxa present in the VV deep samples (1180–1200 cm), *Streptomyces*, is known to produce siderophores allowing for varying degrees of metal tolerance [60]. In the presence of Al, there is also an increase in plant exudate production. These plant exudates can also bind insoluble nutrients, and in turn prevent soil particles from adsorbing P, resulting in an increase in P in solution, which is beneficial to both microbial and plant growth [59]. Similarly, N and S concentrations can indirectly act on microbial communities through changes in vegetation composition and cover [6,12]. Microbial C mineralization and assimilation are strongly limited by P and N. An increase in input of P, e.g., through leaching of fertilizers, may stimulate microbial activity, resulting in a change in microbial community structure [62].

Humification and Fe concentration were found to be the main drivers for the fungal communities at the different sites. Humification is the continuous complex transformation of humic acids in soil and peat environments. These humic acids play key roles in peatlands, including the regulation of redox processes and controlling the mobility, bioavailability, and chemical speciation of micronutrients. In the case of fungi, humic acids can be used as an energy source and their decomposition can result in the release of micronutrients in the environment [63]. The most abundant fungal taxa detected in VV included both ascomycetes (*Candida, Epicoccum, Trichoderma*) and basidiomycetes (*Cryptococcus, Leucosporidiales, Rhodosporidium, Tremella*), which according to FUNGuild classification include saprotrophs (soil, wood, undefined). Saprotrophic fungi are well known for their ability to decompose complex polymers, and it is therefore not surprising that humification is a primary driver for this fungal community [4]. In the acidic, waterlogged (hypoxic) environment of the GK peatland, it is expected that much of the Fe^3+^ would be reduced to Fe^2+^, which is more bioavailable than the oxidized form and can be toxic in high concentrations [64]. In contrast, the higher pH in the NR peatland would theoretically protect the biota from Fe^2+^ toxicity. An evaluation of the most abundant fungal taxa of the two environments showed the presence of endophytic (*Diaportharles, Venturiaceae*) and ectomycorrhizal (*Chroogomphus, Cortinarius, Tricholomataceae*) fungi in the GK samples, versus mostly saprotrophic fungi (*Acremonium, Epicoccum, Geoglossum, Lulwoana, Phoma, Trichocomaceae*) in the NR samples, again reflecting the ‘young’ status of the latter wetland system. Endophytes and ectomycorrhizal fungi are known to produce siderophores and other compounds to the benefit of their plant host. Siderophores can scavenge, regulate, and transport Fe into the cell, depriving competing microorganisms, including plant pathogens, of the essential micronutrient [46,61].

Overall, the LINKTREE results clearly demonstrated that the abiotic parameters that drive community profiles of different microbial groups in peatlands can be completely different. This can also be seen from the different reports in the literature, as summarized by Zhang et al. [45]. It was reported that dissolved organic C, dissolved organic N, and ammonia concentrations—and not pH—were the main drivers for the fungal taxa (*Ascomycota, Basidiomycota*, and *Zygomycota*) reported for bog and fen sites in the peatlands of northwestern Minnesota [65]. The same dominant taxa were also reported for ombrotrophic peatlands in northern England, but the community structure was affected by organic matter, moisture, P, and ammonium concentrations. The fungal community (dominated by *Ascomycota* and *Basidiomycota*) of the boreal peatlands in Finland was mostly affected by the dominant tree species as well as Ca, P, and Fe availability [66]. In contrast, it was reported that fungal communities in ombrotrophic peatlands in southern Canada were driven by pH and Mg concentration [45]. In the study by Zhang et al. [45], which focused on ancient peatlands in the Sanjiang Plain, China, it was shown that the fungal community structure was mostly driven by pH, while in peatlands of the Zoige Plateau, the actinobacterial and fungal community structures were affected by vegetation composition [8].

## 5. Conclusions

The work reported here has provided some insight into the differences in microbial (actinobacterial and fungal) community structures of a developing peatland (NR) and an unimpacted peatland (VV), and how human activities can change a peatland’s microbial community structure (GK). As indicated in Section 4, different physicochemical parameters have been identified to drive microbial community structures in different peatlands around the world. In this study, we found that the actinobacterial community structure was driven by Al, P, and K, while the fungal community structures were driven by humification and Fe levels. Since this study is the first of its kind, the data can be used as a baseline for continuous monitoring of the microbial communities at these sites, providing guidance as to whether anthropomorphic activities will prevent NR from developing into a peatland, whether rehabilitation of GK would drive changes in the microbial community structure more towards that of the VV site, and whether natural changes in the environment (e.g., due to climate change) are causing any changes in the microbial community structure of the unimpacted peatland (VV). Actinobacterial and fungal communities can be used as bioindicators for any changes occurring within these peatlands, whether due to anthropomorphic activities or due to natural events. However, there is clearly a need for further studies to be performed on peatlands located in South Africa—this will not only serve as a guide to the preservation and/or rehabilitation of these sites but will also provide insights into the microbial diversity associated with these sites, which microbial communities should be selected as bioindicators, and how they compare to the well-studied peatlands of the Northern Hemisphere.

## Figures and Tables

**Table 1:** Representative actinobacterial taxa in peat samples as determined by indicator species analysis. Taxa with the highest frequency for each site are highlighted. o = order; f = family; g = genus; NR = Nuwejaars River system (developing peatland); GK = Goukou River system (impacted peatland); VV = Vankervelsvlei (undisturbed peatland).

Taxa	Site	Indicator Value	*p*-Value	Frequency
o__*Acidimicrobiales*.f__C111.g__unidentified	NR	1	0.001	6
o__*Acidimicrobiales*.f__koll13.g__unidentified	NR	1	0.001	6
o__*Acidimicrobiales*.f__AKIW874.g__unidentified	NR	0.993	0.001	9
o__*Actinomycetales*.f__*Intrasporangiaceae*.Other	NR	0.991	0.001	8
o__*Acidimicrobiales*.f__EB1017.g__unidentified	NR	0.986	0.001	8
**o__0319.7L14.f__unidentified.g__unidentified**	**NR**	**0.871**	**0.001**	**18**
o__*Actinomycetales*.f__*Mycobacteriaceae*.g__*Mycobacterium*	NR	0.851	0.001	12
o__*Actinomycetales*.f__*Intrasporangiaceae*.g__*Janibacter*	NR	0.833	0.001	5
o__*Actinomycetales*.f__*Frankiaceae*.g__*Frankia*	NR	0.823	0.001	6
**o__*Gaiellales.*f__*Gaiellaceae.*g__unidentified**	**NR**	**0.729**	**0.001**	**18**
o__*Actinomycetales*.f__*Micromonosporaceae.*g__unidentified	NR	0.677	0.002	7
o__*Acidimicrobiales*.f__TK06.g__unidentified	NR	0.667	0.002	4
o__*Actinomycetales*.f__*Pseudonocardiaceae*.g__unidentified	NR	0.667	0.001	4
o__*Nitriliruptorales*.f__*Nitriliruptoraceae*.g__unidentified	NR	0.667	0.003	4
o__*Rubrobacterales*.f__*Rubrobacteraceae.*g__unidentified	NR	0.667	0.001	4
o__*Actinomycetales*.f__*Nocardioidaceae*.g__unidentified	NR	0.570	0.019	9
o__*Actinomycetales.*f__*Cellulomonadaceae*.g__*Demequina*	NR	0.5	0.007	3
o__*Actinomycetales*.f__*Intrasporangiaceae*.g__*Knoellia*	NR	0.5	0.003	3
o__*Actinomycetales*.f__*Micromonosporaceae*.g__*Catellatospora*	NR	0.5	0.007	3
o__*Actinomycetales*.f__*Micromonosporaceae*.g__*Pilimelia*	NR	0.5	0.008	3
o__*Micrococcales*.f__unidentified.g__unidentified	NR	0.5	0.009	3
o__*Euzebyales.*f__*Euzebyaceae*.g__*Euzebya*	NR	0.5	0.009	3
o__*Actinomycetales.*Other.Other	NR	0.447	0.033	8
o__*Acidimicrobiales*.f__*Iamiaceae.*g__*Iamia*	NR	0.333	0.038	2
o__*Actinomycetales*.f__*Cellulomonadaceae*.g__*Actinotalea*	NR	0.333	0.043	2
o__*Actinomycetales*.f__*Cryptosporangiaceae*.g__unidentified	NR	0.333	0.043	2
o__*Actinomycetales*.f__*Frankiaceae*.Other	NR	0.333	0.048	2
o__*Actinomycetales*.f__*Micromonosporaceae*.g__*Dactylosporangium*	NR	0.333	0.041	2
o__*Actinomycetales*.f__*Pseudonocardiaceae.*Other	NR	0.333	0.039	2
o__*Solirubrobacterales*.f__*Conexibacteraceae*.g__*Conexibacter*	GK	0.993	0.001	13
**o__*Acidimicrobiales*.f__unidentified.g__unidentified**	**GK**	**0.965**	**0.001**	**21**
o__*Actinomycetales*.f__unidentified.g_unidentified	GK	0.913	0.001	19
o__*Solirubrobacterales*.f__*Conexibacteraceae*.g_unidentified	GK	0.849	0.001	12
o__*Actinomycetales*.f__*Actinosynnemataceae.*Other	GK	0.775	0.001	8
o__*Solirubrobacterales*.Other.Other	GK	0.667	0.003	6
o__*Actinomycetales.*f__*Actinospicaceae.*g_unidentified	GK	0.664	0.001	7
o__*Solirubrobacterales*.f__unidentified.g_unidentified	GK	0.598	0.015	19
o__*Solirubrobacterales*.f__*Conexibacteraceae*.Other	GK	0.556	0.005	5
o__*Actinomycetales*.f__*Frankiaceae*.g_unidentified	GK	0.505	0.01	7
o__*Actinomycetales*.f__*Micrococcaceae*.g__*Sinomonas*	GK	0.444	0.025	4
o__*Actinomycetales*.f__*Nocardiaceae.*g__*Nocardia*	GK	0.444	0.021	4
o__*Actinomycetales*.f__*Actinosynnemataceae*.g_unidentified	GK	0.333	0.027	3
**o__*Actinomycetales*.f__*Microbacteriaceae*.g__*Frigoribacterium***	**VV**	**0.583**	**0.006**	**7**
o__*Actinomycetales.*f__*Corynebacteriaceae*.g__*Corynebacterium*	VV	0.5	0.017	6
**o__*Actinomycetales*.f__*Propionibacteriaceae*.g__*Propionibacterium***	**VV**	**0.496**	**0.026**	**7**

**Table 2:** Representative fungal taxa in peat samples as determined by indicator species analysis. All were assigned to the phylum *Ascomycota*. Taxa with the highest frequency for each site are highlighted. o = order; f = family; g = genus; NR = Nuwejaars River system (developing peatland); GK = Goukou River system (impacted peatland); VV = Vankervelsvlei (undisturbed peatland).

**Taxa**	**Site**	**Indicator Value**	***p*** **-Value**	**Frequency**
**o__*Eurotiales*.f__*Trichocomaceae*.g__*Penicillium***	**NR**	**0.846**	**0.014**	**20**
o__*Pleosporales*.f__*Venturiaceae.*g__unidentified	NR	0.666	0.006	8
**o__*Pleosporales*.f__*Incertae_sedis*.g__*Phoma***	**GK**	**1**	**0.001**	**12**
o__*Eurotiales.*f__*Trichocomaceae*.g__unidentified	GK	0.881	0.002	9
o__*Eurotiales*.f__*Trichocomaceae*.g__*Talaromyces*	GK	0.858	0.001	9
o__*Archaeorhizomycetales*.f__*Archaeorhizomycetaceae*.g__*Archaeorhizomyces*	GK	0.833	0.001	5
o__*Pleosporales*.f__*Venturiaceae*.g__*Anungitopsis*	GK	0.667	0.004	4
**o__*Pleosporales*.f__unidentified.g__unidentified**	**GK**	**0.651**	**0.013**	**12**
o_*_Capnodiales*.f__*Incertae_sedis*.g__*Capnobotryella*	GK	0.333	0.05	2
**o__*Capnodiales*.f__*Mycosphaerellaceae*.g__*Septoria***	**VV**	**0.889**	**0.001**	**8**
**o__*Pleosporales*.f__*Phaeosphaeriaceae*.g__*Ampelomyces***	**VV**	**0.646**	**0.017**	**8**
**o__*Dothideales*.f__*Dothioraceae*.g__*Aureobasidium***	**VV**	**0.619**	**0.017**	**8**

**Table 3:** Results of one-way unordered ANOSIM: R values all sites.

	Nuwejaars River System (NR) Developing Peatland		Goukou River System (GK) Impacted Peatland	
	Actinobacteria	Fungi	Actinobacteria	Fungi
Goukou River system (GK)	0.995 ***	0.762 ***	-	-
Vankervelsvlei (VV; undisturbed peatland)	0.436 ***	0.997 ***	0.650 ***	0.912 ***

Level of significance: *** 0.005 < *p*.

**Table 4:** Results of one-way unordered ANOSIM: R values Vankervelsvlei (VV; undisturbed peatland) for actinobacteria at different depths.

	580–600 cm	780–800 cm	980–1000 cm
780–800 cm	0.111		
980–1000 cm	0.074	0.185	
1180–2000 cm	0.889 *	0.704 *	0.889 *

Level of significance: * 0.05 > *p* ≥ 0.01.

**Table 5:** Assignment of shared fungal OTUs to specific fungal trophic modes as determined using FUNGuild.

Trophic Mode	Ascomycete Taxon	Basidiomycete Taxon
Pathotroph–Saprotroph–Symbiotroph	*Acremonium, Alternaria, Aureobasidium, Candida, Epicoccum, Fusarium, Phoma, Trichoderma*	*Agaricales, Cryptococcus*
Pathotroph–Saprotroph	*Pleospora, Teratosphaeriaceae*	*Rhodotorula*
Pathotroph–Symbiotroph	*Tricholomataceae*	-
Saprotroph–Symbiotroph	*Ampelomyces*, *Myxotrichaceae*	-
Saprotroph	*Eupenicillium, Lulwoana, Penicillium*	*Termitomyces*
Pathotroph	*Haematonectria, Lecanicillium*, *Lewia, Metarhizium*	-
Symbiotroph	*Lecythophora, Tuberaceae*	*Cortinarius*

**Table 6:** Assignment of abundant fungal taxa from all three sampling sites to specific fungal guilds as determined using FUNGuild; GK = Goukou River system (impacted peatland); NR = Nuwejaars River system (developing peatland); VV = Vankervelsvlei (undisturbed peatland).

Sampling Site	Abundant Fungal Taxa	Fungal Phylum, Order	Fungal Guild
GK	*Beauveria*	*Ascomycota, Sordariomycetes*	Animal pathogen
	*Chroogomphus*	*Basidiomycota, Agaricomycetes*	Ectomycorrhizal, fungal parasite
	*Cortinarius*	*Basidiomycota, Agaricomycetes*	Ectomycorrhizal
	*Diaportharles*	*Ascomycota, Sordariomycetes*	Endophyte, plant pathogen, undefined saprotroph
	*Penicillium*	*Ascomycota*, *Eurotiomycetes*	Dung saprotroph, undefined saprotroph, wood saprotroph
	*Trichocomaceae*	*Ascomycota*, *Eurotiomycetes*	Saprotroph
	*Tricholomataceae*	*Basidiomycota* *Agaricomycetes*	Ectomycorrhizal, fungal parasite
	*Venturiaceae*	*Ascomycota, Dothideomycetes*	Endophyte, plant pathogen, undefined saprotroph
NR	*Acremonium*	*Ascomycota, Sordariomycetes*	Animal pathogen, endophyte, fungal parasite, plant pathogen, wood saprotroph
	*Epicoccum*	*Ascomycota, Dothideomycetes*	Endophyte, fungal parasite, lichen parasite, plant pathogen, wood saprotroph
	*Geoglossum*	*Ascomycota*, *Leotiomycetes*	Undefined saprotroph
	*Haematonectria*	*Ascomycota, Sodariomycetes*	Plant pathogen
	*Lulwoana*	*Ascomycota, Sordariomycetes*	Undefined saprotroph
	*Phoma*	*Ascomycota, Dothideomycetes*	Endophyte, dung saprotroph, lichen parasite, litter saprotroph, plant pathogen, soil saprotroph, wood saprotroph
	*Trichocomaceae*	*Ascomycota, Eurotiomycetes*	Saprotroph
VV	*Candida*	*Ascomycota, Saccharomycetes*	Animal pathogen, endophyte, epiphyte, endosymbiont, soil saprotroph, undefined saprotroph
	*Cryptococcus*	*Basidiomycota, Tremellomycetes*	Animal pathogen, endophyte, epiphyte, undefined saprotroph
	*Epicoccum*	*Ascomycota, Dothideomycetes*	Endophyte, fungal parasite, lichen parasite, plant pathogen, wood saprotroph
	*Leucosporidiales*	*Basidiomycota, Microbotryomycetes*	Soil saprotroph, undefined saprotroph
	*Rhodosporidium*	*Basidiomycota*, *Microbotryomycetes*	Undefined saprotroph
	*Tremella*	*Basidiomycota*, *Tremellomycetes*	Saprotroph
	*Trichoderma*	*Ascomycota*, *Sordariomycetes*	Animal pathogen, endophyte, epiphyte, fungal parasite, plant pathogen, wood saprotroph

## Data Availability

The data presented in this study are publicly available on the CPUT Esango Research Data Platform and may be accessed with the following link: https://doi.org/10.25381/cput.17206676.v1 (accessed on 17 October 2022). Raw sequence data obtained in this study have been deposited in the National Center for Biotechnology Information Database under BioProject PRJNA805212.

## Supplementary Materials

The following supporting information can be downloaded at: https://www.mdpi.com/article/10.3390/microorganisms10112103/s1, Supplementary Information: QIIME log files. Table S1: Physicochemical parameters recorded for the three study sites and their respective subsites, with a focus on pH, humification, and loss on ignition (LOI). NR: Nuwejaars River system (developing peatland); GK: Goukou River system (impacted peatland); VV: Vankervelsvlei (undisturbed peatland). Table S2: Physicochemical parameters determined for the three sampling sites and respective subsites, with a focus on elemental analyses. All concentrations are provided in mg/kg; NR: Nuwejaars River system (developing peatland); GK: Goukou River system (impacted peatland); VV: Vankervelsvlei (undisturbed peatland); R scripts: Actinobacterial diversity and labdsv analyses; R scripts: Fungal diversity and labdsv analyses. Figure S1: Shade plots of relative abundance of square root transformed relative abundance data of fungal phyla (A) and actinobacterial orders (B) in the peat samples collected from the three study sites. NR = Nuwejaars River system (developing peatland); GK = Goukou River system (impacted peatland); VV = Vankervelsvlei (undisturbed peatland). Top: samples from 0 to 5 cm depth; middle: samples from 25 cm depth; bottom: samples from 50 cm depth. Figure S2: Shade plots of square root transformed relative abundance fungal data of Ascomycota (A) and Basidiomycota (B) classes in the peat samples collected from the three study sites. NR = Nuwejaars River system (developing peatland); GK = Goukou River system (impacted peatland); VV = Vankervelsvlei (undisturbed peatland). Top: samples from 0 to 5 cm depth; middle: samples from 25 cm depth; bottom: samples from 50 cm depth. Figure S3: Dendogram of cluster analysis (group average linkage) of Bray–Curtis similarity of square root transformed actinobacterial relative abundance data from samples from the three study sites. NR = Nuwejaars River system (developing peatland); GK = Goukou River system (impacted peatland); VV = Vankervelsvlei (undisturbed peatland). Top: samples from 0 to 5 cm depth; middle: samples from 25 cm depth; bottom: samples from 50 cm depth. Figure S4: Dendogram of cluster analysis (group average linkage) of Bray–Curtis similarity of square root transformed fungal relative abundance data from samples from the three study sites. NR = Nuwejaars River system (developing peatland); GK = Goukou River system (impacted peatland); VV = Vankervelsvlei (undisturbed peatland). Top: samples from 0 to 5 cm depth; middle: samples from 25 cm depth; bottom: samples from 50 cm depth; Supplementary information_otu_table.guilds_matched; Supplementary information_actinobacterial and fungal taxa.
