# Supplementary material for: Impact of Physicochemical Parameters on the Diversity and Distribution of Microbial Communities Associated with Three South African Peatlands
Source: Microorganisms. 2022 Oct 23;10(11):2103. doi: 10.3390/microorganisms10112103 (PMC9694404; doi:10.3390/microorganisms10112103)
Supplement: Supplementary file 1 [file microorganisms-10-02103-s001.zip › Weels et al_MS_MDPI_Supplementary.pdf]

Impact of physicochemical parameters on the diversity and distribution of microbial communities associated with three South African peatlands

Shandré S. L. Weels [1,2], Pamela J. Welz[1], Alaric Prins[1], and Marilize Le Roes-Hill[1]

[1] Applied Microbial and Health Biotechnology Institute, Cape Peninsula University of Technology, Bellville Campus, PO Box 1906, Bellville, South Africa, 7530
[2] Department of Environmental and Occupational Studies, Cape Peninsula University of Technology, District Six Campus, PO Box 652, Cape Town, South Africa, 8000
* Correspondence: leroesm@cput.ac.za; Tel.: +27 (21) 953 8499

Supplementary information

Physicochemical data

Table S1: Physicochemical parameters recorded for the three study sites and their respective subsites, with a focus on pH, humification, and loss on ignition (LOI). NR: Nuwejaars River system (developing peatland); GK: Goukou River system (impacted peatland); VV: Vankervelsvlei (undisturbed peatland).

	pH	Humification	LOI
NR top	7.12	0.058	10807.35
NR middle	7.08	0.043	6071.61
NR bottom	6.43	0.031	3777.17
NR top	7.08	0.056	17914.14
NR middle	7.08	0.039	7426.08
NR bottom	6.43	0.146	7521.60
GK top	2.93	0.174	19061.42
GK middle	2.99	0.07	9832.87
GK bottom	2.99	0.05	7712.59
GK top	3.28	0.053	7068.00
GK middle	3.31	0.079	7876.68
GK bottom	3.23	0.121	9554.92
GK top	3.07	0.135	12250.18
GK middle	3.03	0.114	12486.23
GK bottom	3.17	0.054	6381.55
VV 580-600 cm	3.96	0.571	97198.61
VV 780-800 cm	4.14	0.798	97610.48
VV 980-1000 cm	4.25	0.655	95903.20
VV 1180-1200 cm	4.51	0.064	12047.72
VV 580-600 cm	4.49	0.201	97380.21
VV 780-800 cm	4.16	0.324	92923.11
VV 980-1000 cm	4.24	0.429	92926.49
VV 1180-1200 cm	4.56	0.05	8293.92
VV 580-600 cm	4.1	0.534	95641.33
VV 780-800 cm	4.48	0.201	91916.50
VV 980-1000 cm	4.36	0.31	41349.44
VV 1180-1200 cm	4.64	0.076	11259.04

Table S2: Physicochemical parameters determined for the three sampling sites and respective subsites, with a focus on elemental analyses. All concentrations are provided in mg/kg; NR: Nuwejaars River system (developing peatland); GK: Goukou River system (impacted peatland); VV: Vankervelsvlei (undisturbed peatland).

	N	C	S	Al	Ca	Cr	Fe	K	Mg	Mn	Na	P	Si	Ti
NR top	200	2670	40.76	1609.85	242.23	0.00	698.67	333.17	306.56	6.91	337.48	22.97	38507.80	192.50
NR middle	70	1480	16.05	1973.74	174.54	6.43	1011.68	428.83	385.14	21.82	125.43	16.13	40192.40	202.72
NR bottom	50	850	22.76	1415.87	96.28	6.58	814.31	303.52	324.93	14.90	142.78	12.39	42186.13	161.52
NR top	250	2750	336.24	4688.04	404.80	5.62	2531.82	974.39	697.92	6.36	535.92	28.19	30340.57	260.82
NR middle	90	1750	81.89	2415.67	244.80	6.33	1236.65	545.60	480.07	7.17	233.51	15.90	38855.61	238.64
NR bottom	80	1430	91.82	2388.70	211.50	0.00	1274.18	537.36	524.19	7.16	260.72	15.88	38880.35	216.22
GK top	250	4590	22.11	746.31	42.88	0.00	209.82	99.61	54.27	7.75	29.68	8.59	37508.85	263.78
GK middle	150	2440	30.82	439.32	21.44	0.00	97.92	66.41	36.18	7.75	0.00	8.59	41752.84	167.86
GK bottom	60	2390	26.42	545.18	14.29	0.00	76.93	124.52	36.18	0.00	0.00	4.29	42547.42	113.91
GK top	60	2140	3.61	1063.89	14.29	0.00	83.93	182.62	48.24	0.00	0.00	8.59	42238.94	173.86
GK middle	110	2720	6.97	1122.12	14.29	0.00	90.92	190.92	48.24	7.75	0.00	8.59	41888.39	191.84
GK bottom	90	2770	31.02	968.62	14.29	0.00	76.93	149.42	48.24	0.00	0.00	8.59	41477.08	197.84
GK top	140	4120	6.86	762.19	14.29	0.00	62.95	107.91	36.18	0.00	7.42	8.59	40411.40	215.82
GK middle	200	3240	18.07	1037.43	14.29	0.00	90.92	149.42	48.24	0.00	0.00	8.59	40023.46	209.83
GK bottom	60	2500	15.67	756.90	7.15	0.00	69.94	132.82	36.18	0.00	0.00	8.59	42949.39	137.89
VV 580-600 cm	1330	52902	717	275.37	257.42	0.00	83.97	33.22	217.19	0.00	19.79	11.45	589.06	47.98
VV 780-800 cm	943	54901	461	261.02	352.45	0.00	108.92	32.32	289.54	0.00	28.88	11.14	274.99	38.90
VV 980-1000 cm	954	53908	753	408.38	503.48	11.48	211.16	41.77	384.33	0.00	87.11	14.40	627.98	60.33
VV 1180-1200 cm	199	3733	68	7676.61	176.01	6.02	1728.54	1270.36	403.07	0.00	508.96	41.53	31049.55	448.18
VV 580-600 cm	1285	54947	667	700.08	304.93	0.00	149.20	70.83	308.73	0.00	0.00	18.32	0.00	127.89
VV 780-800 cm	1568	52274	593	396.81	475.53	0.00	144.19	54.45	356.00	0.00	90.38	12.07	2084.52	78.65
VV 980-1000 cm	1462	52929	803	360.67	538.65	0.00	223.85	34.28	379.76	0.00	76.60	13.30	2034.24	55.71
VV 1180-1200 cm	143	2354	36	6085.06	163.86	6.27	1199.40	898.28	270.96	7.10	496.67	23.62	34556.08	648.74
VV 580-600 cm	1410	54385	818	292.17	402.25	0.00	98.41	35.94	300.22	8.38	64.24	13.94	1077.30	45.42
VV 780-800 cm	959	54406	551	668.19	519.47	0.00	187.29	63.51	369.08	0.00	0.00	16.42	2238.01	183.47
VV 980-1000 cm	1123	27041	544	2874.65	314.38	4.01	471.73	540.41	251.10	4.54	404.67	32.73	23084.72	355.13
VV 1180-1200 cm	168	3498	47	6247.09	158.56	6.07	1216.48	935.53	278.26	6.87	375.27	22.86	33335.49	457.52

R scripts: Actinobacterial diversity and labdsv analyses

```
library(phyloseq)
library(ggplot2)
#Import OTU table and tree
otutable <- import_biom(BIOMfilename = 'otu_16s_json.biom',
                        treefilename = 'rep_set.tre',
                        parseFunction = parse_taxonomy_default)
#import mapping file
mapping <- import_qiime_sample_data(mapfilename = 'map_16S.txt')
#Merge OTU table and map into one phyloseq object
phylo <- merge_phyloseq(otutable, mapping)
#Remove zero sum OTUs
phylo = prune_taxa(taxa_sums(phylo) > 0, phylo)
#Label rank names for 7 ranks - kingdom to species
colnames(tax_table(phylo)) <- c("Kingdom", "Phylum", "Class", "Order", "Family", "Genus", "Species")
#Check sample + OTU count
phylo
#Check taxa rank assignments
rank_names(phylo)
#Filter Actinobacteria from Phylum level
SUBS <- subset_taxa(phylo, Phylum=="p__Actinobacteria")
#DiversityIndices
plot_richness(SUBS, measures=c("Observed"))
plot_richness(SUBS, measures=c("Chao1"))
plot_richness(SUBS, measures=c("Shannon"))
library(labdsv)
#all sites
spec <- read.csv("labdsv_actino_species.csv", dec = ";", row.names = 1)
group <- read.csv("labdsv_actino_group.csv", dec = ",", row.names = 1)
spe.only <- spec[,3:90]
View(spe.only)
spe.only[ , !apply(spe.only==0,2,all)]
(iva <- indval(spe.only, group$id))
gr <- iva$maxcls[iva$pval <= 0.05]
iv <- iva$indcls[iva$pval <= 0.05]
pv <- iva$pval[iva$pval <= 0.05]
fr <- apply(spe.only > 0, 2, sum)[iva$pval <= 0.05]
fidg <- data.frame(group=gr, indval=iv, pvalue=pv, freq=fr)
fidg <- fidg[order(fidg$group, -fidg$indval),]
fidg
write.csv(fidg, "indval-actino_by_id.csv")
```

R scripts: Fungal diversity and labdsv analyses

```
#Import OTU table and tree
otutable <- import_biom(BIOMfilename = 'otu_its_json.biom',
                        parseFunction = parse_taxonomy_default)
#import mapping file
mapping <- import_qiime_sample_data(mapfilename = 'map_ITS.txt')
#Merge OTU table and map into one phyloseq object
```

```
phylo <- merge_phyloseq(otutable, mapping)
#Remove zero sum OTUs
phylo = prune_taxa(taxa_sums(phylo) > 0, phylo)
#Label rank names for 7 ranks - kingdom to species
colnames(tax_table(phylo)) <- c("Kingdom", "Phylum", "Class", "Order", "Family", "Genus", "Species")
#Check sample + OTU count
phylo
#Check taxa rank assignments
rank_names(phylo)
#DiversityIndices
plot_richness(phylo, measures=c("Observed"))
plot_richness(phylo, measures=c("Chao1"))
plot_richness(phylo, measures=c("Shannon"))
#allsites
library(labdsv)
#all sites
spec <- read.csv("labdsv_fungi_species.csv", dec = ";", row.names = 1)
group <- read.csv("labdsv_fungi_group.csv", dec = ",", row.names = 1)
spe.only <- spec[,3:90]
View(spe.only)
spe.only[ , !apply(spe.only==0,2,all)]
(iva <- indval(spe.only, group$id))
gr <- iva$maxcls[iva$pval <= 0.05]
iv <- iva$indcls[iva$pval <= 0.05]
pv <- iva$pval[iva$pval <= 0.05]
fr <- apply(spe.only > 0, 2, sum)[iva$pval <= 0.05]
fidg <- data.frame(group=gr, indval=iv, pvalue=pv, freq=fr)
fidg <- fidg[order(fidg$group, -fidg$indval),]
fidg
write.csv(fidg, "indval_fungi_by_id.csv")
```

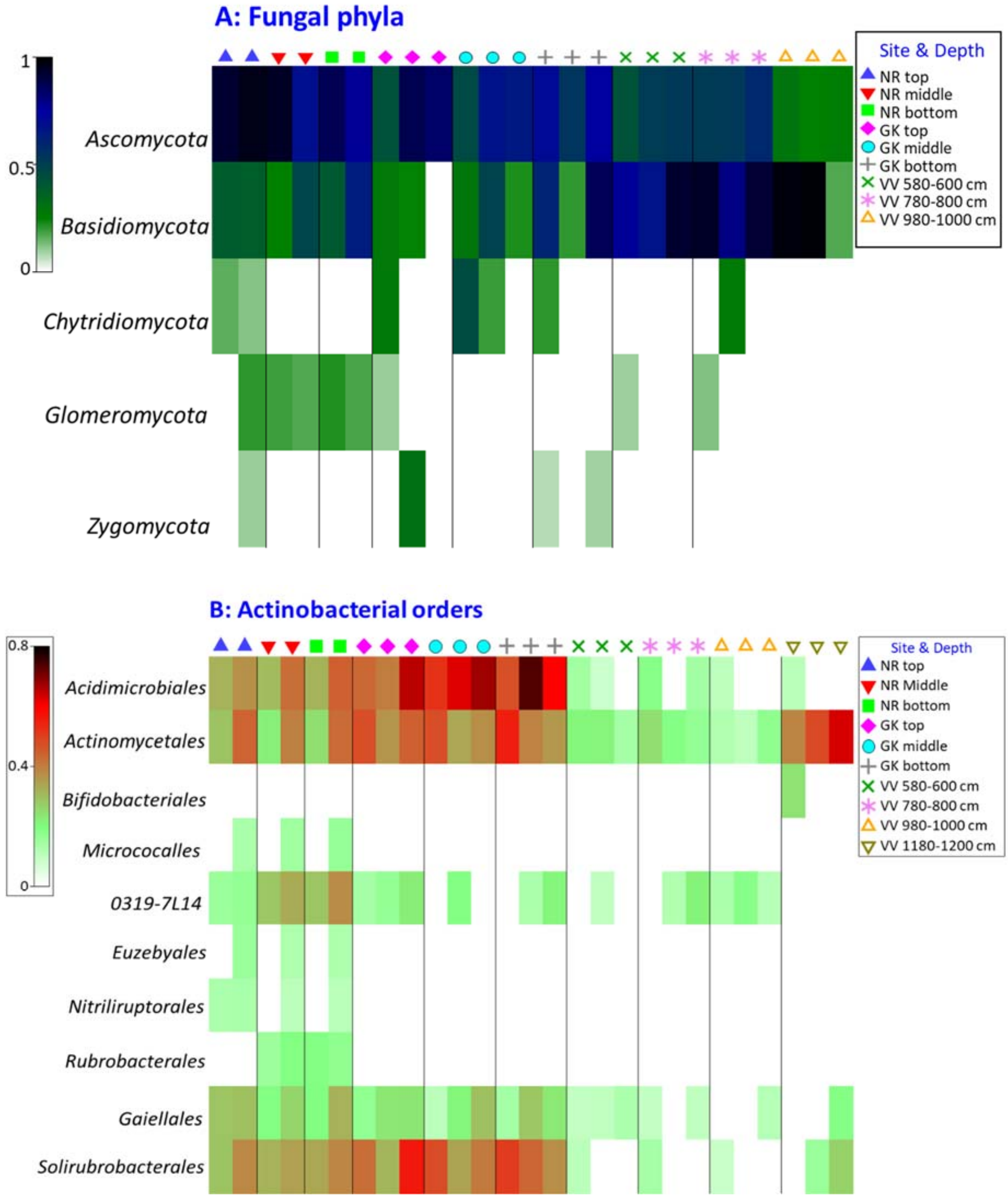

Figure S1: Shade plots of relative abundance of square root transformed relative abundance data of fungal phyla (A) and actinobacterial orders (B) in the peat samples collected from the three study sites. NR = Nuwejaars River system (developing peatland); GK = Goukou River system (impacted peatland); VV = Vankervelsvlei (undisturbed peatland). Top: 0 to -5 cm; Middle: samples from a 25 cm depth; Bottom: samples from a 50 cm depth.

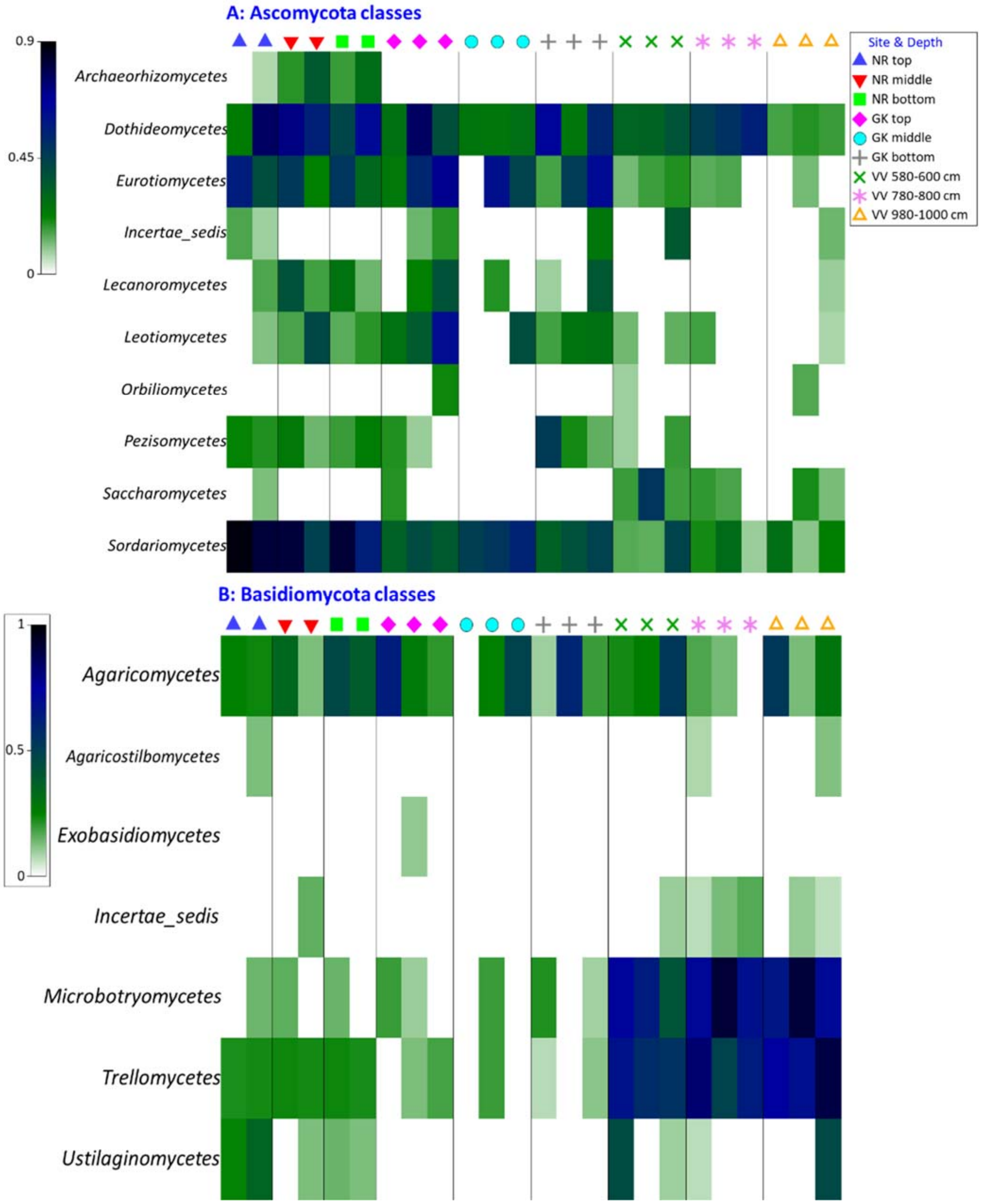

Figure S2: Shade plots of square root transformed relative abundance fungal data of *Ascomycota* (A) and *Basidiomycota* (B) classes in the peat samples collected from the three study sites. NR = Nuwejaars River system (developing peatland); GK = Goukou River system (impacted peatland); VV = Vankervelsvlei (undisturbed peatland). Top: 0 to -5 cm; Middle: samples from a 25 cm depth; Bottom: samples from a 50 cm depth.

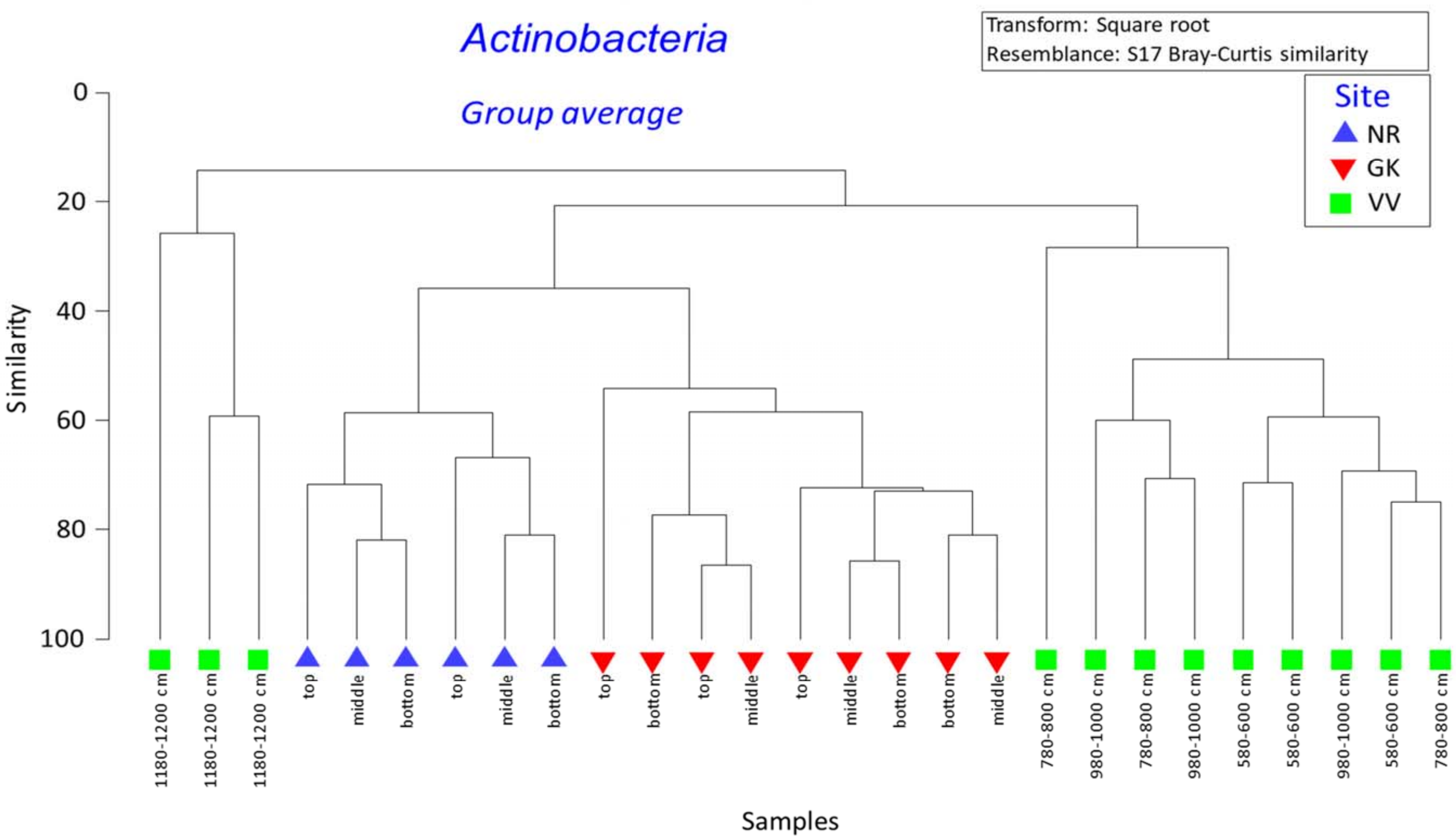

Figure S3: Dendogram of cluster analysis (group average linkage) of Bray-Curtis similarity of square root transformed actinobacterial relative abundance data from samples from the three study sites. NR = Nuwejaars River system (developing peatland); GK = Goukou River system (impacted peatland); VV = Vankervelsvlei (undisturbed peatland). Top: 0 to -5 cm; Middle: samples from a 25 cm depth; Bottom: samples from a 50 cm depth.

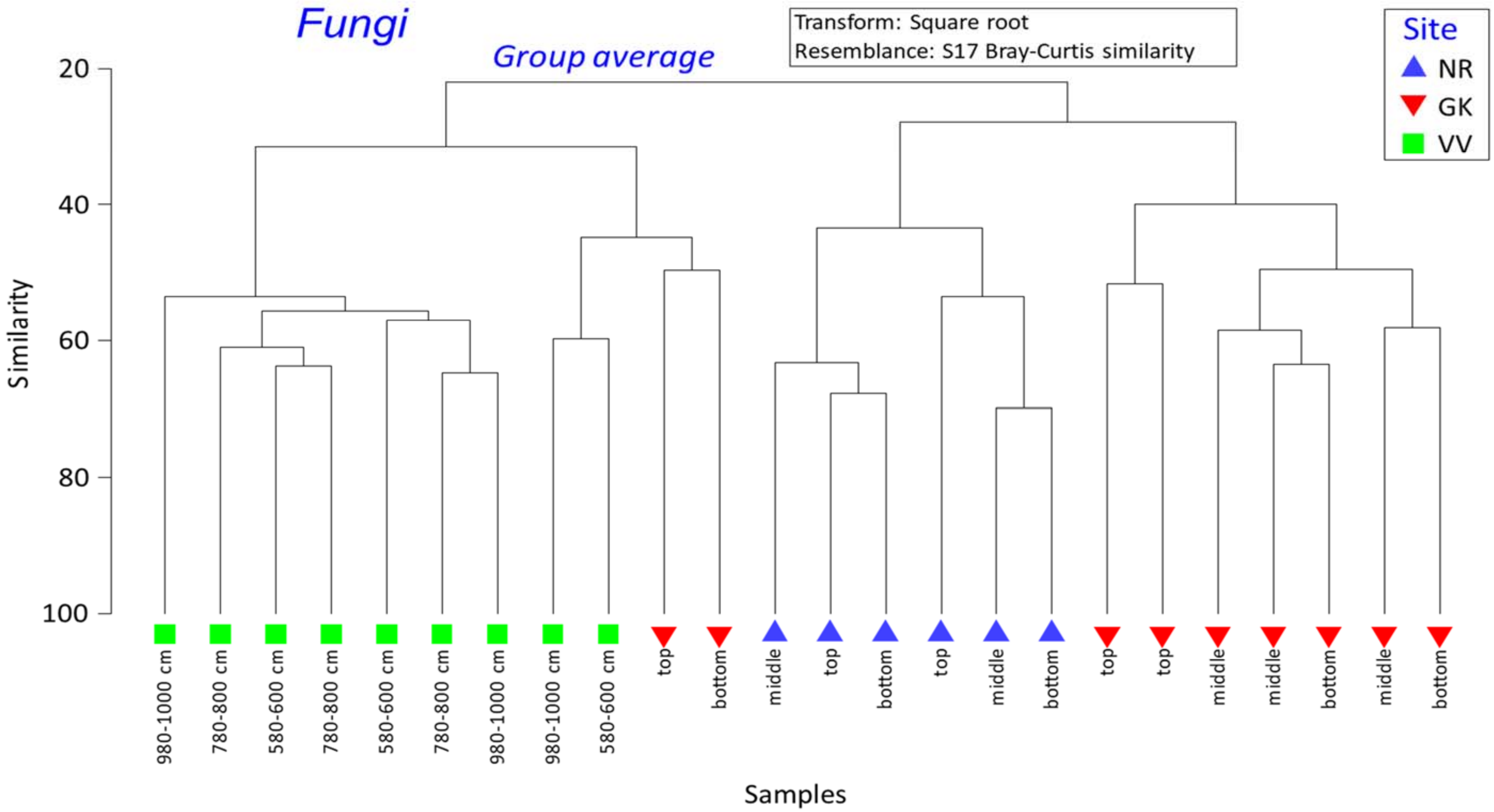

Figure S4: Dendogram of cluster analysis (group average linkage) of Bray-Curtis similarity of square root transformed fungal relative abundance data from samples from the three study sites. NR = Nuwejaars River system (developing peatland); GK = Goukou River system (impacted peatland); VV = Vankervelsvlei (undisturbed peatland). Top: 0 to -5 cm; Middle: samples from a 25 cm depth; Bottom: samples from a 50 cm depth.
