# Supplementary material for: Impact of Physicochemical Parameters on the Diversity and Distribution of Microbial Communities Associated with Three South African Peatlands
Source: Microorganisms. 2022 Oct 23;10(11):2103. doi: 10.3390/microorganisms10112103 (PMC9694404; doi:10.3390/microorganisms10112103)
Supplement: Supplementary file 1 [file microorganisms-10-02103-s001.zip › Weels et al_MS_QIIMELogs_Supplementary.pdf]

QIIME analyses log - 16S

Input file paths
Sequence read filepath: 1A_read1.fastq.gz (md5: 5ad6d7a0a1cc9a94bdec84cf9ea86553)
Quality filter results
Total number of input sequences: 26114
Barcode not in mapping file: 0
Read too short after quality truncation: 105
Count of N characters exceeds limit: 0
Illumina quality digit = 0: 0
Barcode errors exceed max: 0

Result summary (after quality filtering)
Median sequence length: 301.00
1A 26009

Total number seqs written 26009

Input file paths
Sequence read filepath: 1A_read2.fastq.gz (md5: 93fe1bd1d58861c2ffc36892d12977ad)
Quality filter results
Total number of input sequences: 26114
Barcode not in mapping file: 0
Read too short after quality truncation: 252
Count of N characters exceeds limit: 0
Illumina quality digit = 0: 0
Barcode errors exceed max: 0

Result summary (after quality filtering)
Median sequence length: 301.00
1A 25862

Total number seqs written 25862

Input file paths
Sequence read filepath: 1B_read1.fastq.gz (md5: 948ed3e51f3a823d5aef104bd8121b2e)
Quality filter results
Total number of input sequences: 35417

Barcode not in mapping file: 0
Read too short after quality truncation: 161
Count of N characters exceeds limit: 0
Illumina quality digit = 0: 0
Barcode errors exceed max: 0

Result summary (after quality filtering)
Median sequence length: 301.00
1B	35256

Total number seqs written 35256

Input file paths
Sequence read filepath: 1B_read2.fastq.gz (md5: 1e5fc0afb2a8f5d142d5f6ce8336e203)
Quality filter results
Total number of input sequences: 35417
Barcode not in mapping file: 0
Read too short after quality truncation: 138
Count of N characters exceeds limit: 0
Illumina quality digit = 0: 0
Barcode errors exceed max: 0

Result summary (after quality filtering)
Median sequence length: 301.00
1B	35279

Total number seqs written 35279

Input file paths
Sequence read filepath: 1C_read1.fastq.gz (md5: a95b3286a3343a85f484fb5b5f23e2df)
Quality filter results
Total number of input sequences: 46051
Barcode not in mapping file: 0
Read too short after quality truncation: 299
Count of N characters exceeds limit: 0
Illumina quality digit = 0: 0
Barcode errors exceed max: 0

Result summary (after quality filtering)
Median sequence length: 301.00
1C	45752

Total number seqs written 45752

Input file paths
Sequence read filepath: 1C_read2.fastq.gz (md5: 4a479a4e41b2b0cfa093f48bbf6e2766)
Quality filter results
Total number of input sequences: 46051
Barcode not in mapping file: 0
Read too short after quality truncation: 261
Count of N characters exceeds limit: 0
Illumina quality digit = 0: 0
Barcode errors exceed max: 0

Result summary (after quality filtering)
Median sequence length: 301.00
1C 45790

Total number seqs written 45790

Input file paths
Sequence read filepath: 2A_read1.fastq.gz (md5: 7dd61c8382bdcd96014576e01922636d)
Quality filter results
Total number of input sequences: 37978
Barcode not in mapping file: 0
Read too short after quality truncation: 253
Count of N characters exceeds limit: 0
Illumina quality digit = 0: 0
Barcode errors exceed max: 0

Result summary (after quality filtering)
Median sequence length: 301.00
2A 37725

Total number seqs written 37725

Input file paths
Sequence read filepath: 2A_read2.fastq.gz (md5: 4a64a2656410d836114e3a18d01b2e02)
Quality filter results

Total number of input sequences: 37978
Barcode not in mapping file: 0
Read too short after quality truncation: 225
Count of N characters exceeds limit: 0
Illumina quality digit = 0: 0
Barcode errors exceed max: 0

Result summary (after quality filtering)
Median sequence length: 301.00
2A 37753

Total number seqs written 37753

Input file paths
Sequence read filepath: 2B_read1.fastq.gz (md5:
b85a58d71276a77966ff8bdbcd9d8a74)
Quality filter results
Total number of input sequences: 48218
Barcode not in mapping file: 0
Read too short after quality truncation: 280
Count of N characters exceeds limit: 0
Illumina quality digit = 0: 0
Barcode errors exceed max: 0

Result summary (after quality filtering)
Median sequence length: 301.00
2B 47938

Total number seqs written 47938

Input file paths
Sequence read filepath: 2B_read2.fastq.gz (md5: ec86bb1abbf7f850efd85f1d6e14e5b9)
Quality filter results
Total number of input sequences: 48218
Barcode not in mapping file: 0
Read too short after quality truncation: 257
Count of N characters exceeds limit: 0
Illumina quality digit = 0: 0
Barcode errors exceed max: 0

Result summary (after quality filtering)

Median sequence length: 301.00
2B 47961

Total number seqs written 47961

Input file paths
Sequence read filepath: 2C_read1.fastq.gz (md5:
1a5cb3ae2b6858039c14f918ad6343c1)
Quality filter results
Total number of input sequences: 45947
Barcode not in mapping file: 0
Read too short after quality truncation: 338
Count of N characters exceeds limit: 0
Illumina quality digit = 0: 0
Barcode errors exceed max: 0

Result summary (after quality filtering)
Median sequence length: 301.00
2C 45609

Total number seqs written 45609

Input file paths
Sequence read filepath: 2C_read2.fastq.gz (md5:
6c692241ce50fddadac5d9c2a1e503d8)
Quality filter results
Total number of input sequences: 45947
Barcode not in mapping file: 0
Read too short after quality truncation: 305
Count of N characters exceeds limit: 0
Illumina quality digit = 0: 0
Barcode errors exceed max: 0

Result summary (after quality filtering)
Median sequence length: 301.00
2C 45642

Total number seqs written 45642

Input file paths

Sequence read filepath: G1_read1.fastq.gz (md5: c136ea509b613cc1635a788738a84db5)
Quality filter results
Total number of input sequences: 39848
Barcode not in mapping file: 0
Read too short after quality truncation: 374
Count of N characters exceeds limit: 0
Illumina quality digit = 0: 0
Barcode errors exceed max: 0

Result summary (after quality filtering)
Median sequence length: 301.00
G1 39474

Total number seqs written 39474

Input file paths
Sequence read filepath: G1_read2.fastq.gz (md5: 1a8d469f05641d8635988eab3c1f1568)
Quality filter results
Total number of input sequences: 39848
Barcode not in mapping file: 0
Read too short after quality truncation: 320
Count of N characters exceeds limit: 0
Illumina quality digit = 0: 0
Barcode errors exceed max: 0

Result summary (after quality filtering)
Median sequence length: 301.00
G1 39528

Total number seqs written 39528

Input file paths
Sequence read filepath: G2_read1.fastq.gz (md5: cf454b5d9a6ad1b130ffb97b8624f5c9)
Quality filter results
Total number of input sequences: 39809
Barcode not in mapping file: 0
Read too short after quality truncation: 315
Count of N characters exceeds limit: 0
Illumina quality digit = 0: 0

Barcode errors exceed max: 0

Result summary (after quality filtering)
Median sequence length: 301.00
G2 39494

Total number seqs written 39494

Input file paths
Sequence read filepath: G2_read2.fastq.gz (md5: 4a70e2f5e296734ffb9670c783ca9c82)
Quality filter results
Total number of input sequences: 39809
Barcode not in mapping file: 0
Read too short after quality truncation: 258
Count of N characters exceeds limit: 0
Illumina quality digit = 0: 0
Barcode errors exceed max: 0

Result summary (after quality filtering)
Median sequence length: 301.00
G2 39551

Total number seqs written 39551

Input file paths
Sequence read filepath: G3_read1.fastq.gz (md5:
6e99eeacdcfd0cb1e662e8b39388e992)
Quality filter results
Total number of input sequences: 28695
Barcode not in mapping file: 0
Read too short after quality truncation: 36
Count of N characters exceeds limit: 20
Illumina quality digit = 0: 0
Barcode errors exceed max: 0

Result summary (after quality filtering)
Median sequence length: 301.00
G3 28639

Total number seqs written 28639

Input file paths
Sequence read filepath: G3_read2.fastq.gz (md5: 8c1263b756ae92fc8fe8ad0029bb44ba)
Quality filter results
Total number of input sequences: 28695
Barcode not in mapping file: 0
Read too short after quality truncation: 25
Count of N characters exceeds limit: 23
Illumina quality digit = 0: 0
Barcode errors exceed max: 0

Result summary (after quality filtering)
Median sequence length: 301.00
G3 28647

Total number seqs written 28647

Input file paths
Sequence read filepath: G4_read1.fastq.gz (md5: 48eb9bd7751aade5a76e1fc9a3502e27)
Quality filter results
Total number of input sequences: 24784
Barcode not in mapping file: 0
Read too short after quality truncation: 544
Count of N characters exceeds limit: 53
Illumina quality digit = 0: 0
Barcode errors exceed max: 0

Result summary (after quality filtering)
Median sequence length: 301.00
G4 24187

Total number seqs written 24187

Input file paths
Sequence read filepath: G4_read2.fastq.gz (md5: f7e260d78cb31d743b773f99ada80176)
Quality filter results
Total number of input sequences: 24784
Barcode not in mapping file: 0

Read too short after quality truncation: 551
Count of N characters exceeds limit: 33
Illumina quality digit = 0: 0
Barcode errors exceed max: 0

Result summary (after quality filtering)
Median sequence length: 300.00
G4 24200

Total number seqs written 24200

Input file paths
Sequence read filepath: G5_read1.fastq.gz (md5: d0b8caf644345f396bda2ae1b0cf4098)
Quality filter results
Total number of input sequences: 17191
Barcode not in mapping file: 0
Read too short after quality truncation: 146
Count of N characters exceeds limit: 0
Illumina quality digit = 0: 0
Barcode errors exceed max: 0

Result summary (after quality filtering)
Median sequence length: 301.00
G5 17045

Total number seqs written 17045

Input file paths
Sequence read filepath: G5_read2.fastq.gz (md5: 4049aca25d20633f1a70e6be91b2a3ae)
Quality filter results
Total number of input sequences: 17191
Barcode not in mapping file: 0
Read too short after quality truncation: 98
Count of N characters exceeds limit: 0
Illumina quality digit = 0: 0
Barcode errors exceed max: 0

Result summary (after quality filtering)
Median sequence length: 301.00

G5	17093

Total number seqs written 17093

Input file paths
Sequence read filepath: G6_read1.fastq.gz (md5: 39b37225230b2c183139795012a731ed)
Quality filter results
Total number of input sequences: 23446
Barcode not in mapping file: 0
Read too short after quality truncation: 217
Count of N characters exceeds limit: 0
Illumina quality digit = 0: 0
Barcode errors exceed max: 0

Result summary (after quality filtering)
Median sequence length: 301.00
G6	23229

Total number seqs written 23229

Input file paths
Sequence read filepath: G6_read2.fastq.gz (md5: 56ded08a83d0e214566b4484ce048173)
Quality filter results
Total number of input sequences: 23446
Barcode not in mapping file: 0
Read too short after quality truncation: 144
Count of N characters exceeds limit: 0
Illumina quality digit = 0: 0
Barcode errors exceed max: 0

Result summary (after quality filtering)
Median sequence length: 301.00
G6	23302

Total number seqs written 23302

Input file paths
Sequence read filepath: G7_read1.fastq.gz (md5: 6e4fcdc08c14e5bf8a24132fc2f4ac41)

Quality filter results
Total number of input sequences: 41361
Barcode not in mapping file: 0
Read too short after quality truncation: 1271
Count of N characters exceeds limit: 30
Illumina quality digit = 0: 0
Barcode errors exceed max: 0

Result summary (after quality filtering)
Median sequence length: 301.00
G7 40060

Total number seqs written 40060

Input file paths
Sequence read filepath: G7_read2.fastq.gz (md5: 90c3f56b03d5dd7bb946162044316bcc)
Quality filter results
Total number of input sequences: 41361
Barcode not in mapping file: 0
Read too short after quality truncation: 1165
Count of N characters exceeds limit: 43
Illumina quality digit = 0: 0
Barcode errors exceed max: 0

Result summary (after quality filtering)
Median sequence length: 301.00
G7 40153

Total number seqs written 40153

Input file paths
Sequence read filepath: G8_read1.fastq.gz (md5: 2a9caed19b6fa44abbe9a7a03c57d31d)
Quality filter results
Total number of input sequences: 37494
Barcode not in mapping file: 0
Read too short after quality truncation: 633
Count of N characters exceeds limit: 32
Illumina quality digit = 0: 0
Barcode errors exceed max: 0

Result summary (after quality filtering)
Median sequence length: 301.00
G8	36829

Total number seqs written	36829

Input file paths
Sequence read filepath: G8_read2.fastq.gz (md5: ad686839c8b23f5d7e60ae4d37f3dd85)
Quality filter results
Total number of input sequences: 37494
Barcode not in mapping file: 0
Read too short after quality truncation: 580
Count of N characters exceeds limit: 42
Illumina quality digit = 0: 0
Barcode errors exceed max: 0

Result summary (after quality filtering)
Median sequence length: 301.00
G8	36872

Total number seqs written	36872

Input file paths
Sequence read filepath: G9_read1.fastq.gz (md5: 10808c096c20989538d74d4b9f52bf6a)
Quality filter results
Total number of input sequences: 7190
Barcode not in mapping file: 0
Read too short after quality truncation: 143
Count of N characters exceeds limit: 7
Illumina quality digit = 0: 0
Barcode errors exceed max: 0

Result summary (after quality filtering)
Median sequence length: 301.00
G9	7040

Total number seqs written	7040

Input file paths
Sequence read filepath: G9_read2.fastq.gz (md5: 418107ce7d48cfd142e5dac7ad4fad2d)
Quality filter results
Total number of input sequences: 7190
Barcode not in mapping file: 0
Read too short after quality truncation: 110
Count of N characters exceeds limit: 12
Illumina quality digit = 0: 0
Barcode errors exceed max: 0

Result summary (after quality filtering)
Median sequence length: 301.00
G9 7068

Total number seqs written 7068

Input file paths
Sequence read filepath: V1_read1.fastq.gz (md5: 7b27af97d0dffd55b21afd024aaa4a82)
Quality filter results
Total number of input sequences: 18397
Barcode not in mapping file: 0
Read too short after quality truncation: 52
Count of N characters exceeds limit: 28
Illumina quality digit = 0: 0
Barcode errors exceed max: 0

Result summary (after quality filtering)
Median sequence length: 301.00
V1 18317

Total number seqs written 18317

Input file paths
Sequence read filepath: V1_read2.fastq.gz (md5: 0f08372147e1ae771db8a01d5aa307f3)
Quality filter results
Total number of input sequences: 18397
Barcode not in mapping file: 0

Read too short after quality truncation: 59
Count of N characters exceeds limit: 16
Illumina quality digit = 0: 0
Barcode errors exceed max: 0

Result summary (after quality filtering)
Median sequence length: 300.00
V1 18322

Total number seqs written 18322

Input file paths
Sequence read filepath: V2_read1.fastq.gz (md5: e7eaab0380317fc35b2b2a4c30e67be0)
Quality filter results
Total number of input sequences: 33288
Barcode not in mapping file: 0
Read too short after quality truncation: 2497
Count of N characters exceeds limit: 21
Illumina quality digit = 0: 0
Barcode errors exceed max: 0

Result summary (after quality filtering)
Median sequence length: 301.00
V2 30770

Total number seqs written 30770

Input file paths
Sequence read filepath: V2_read2.fastq.gz (md5: a2fa88a17efa421688a163168a5c3234)
Quality filter results
Total number of input sequences: 33288
Barcode not in mapping file: 0
Read too short after quality truncation: 2495
Count of N characters exceeds limit: 28
Illumina quality digit = 0: 0
Barcode errors exceed max: 0

Result summary (after quality filtering)
Median sequence length: 301.00
V2 30765

Total number seqs written 30765

Input file paths
Sequence read filepath: V3_read1.fastq.gz (md5: 37b73bda54c37dcd0756bd35dea286f9)
Quality filter results
Total number of input sequences: 59975
Barcode not in mapping file: 0
Read too short after quality truncation: 1041
Count of N characters exceeds limit: 42
Illumina quality digit = 0: 0
Barcode errors exceed max: 0

Result summary (after quality filtering)
Median sequence length: 301.00
V3 58892

Total number seqs written 58892

Input file paths
Sequence read filepath: V3_read2.fastq.gz (md5: cae2ebed09fc20519d659e0d03d9fc27)
Quality filter results
Total number of input sequences: 59975
Barcode not in mapping file: 0
Read too short after quality truncation: 1008
Count of N characters exceeds limit: 58
Illumina quality digit = 0: 0
Barcode errors exceed max: 0

Result summary (after quality filtering)
Median sequence length: 301.00
V3 58909

Total number seqs written 58909

Input file paths
Sequence read filepath: V4_read1.fastq.gz (md5: 3c243e259068e022a36a780950edf0dc)

Quality filter results
Total number of input sequences: 37377
Barcode not in mapping file: 0
Read too short after quality truncation: 10046
Count of N characters exceeds limit: 22
Illumina quality digit = 0: 0
Barcode errors exceed max: 0

Result summary (after quality filtering)
Median sequence length: 301.00
V4 27309

Total number seqs written 27309

Input file paths
Sequence read filepath: V4_read2.fastq.gz (md5: 63640f730f78a54357dc7b6be2f964a6)
Quality filter results
Total number of input sequences: 37377
Barcode not in mapping file: 0
Read too short after quality truncation: 10046
Count of N characters exceeds limit: 30
Illumina quality digit = 0: 0
Barcode errors exceed max: 0

Result summary (after quality filtering)
Median sequence length: 301.00
V4 27301

Total number seqs written 27301

Input file paths
Sequence read filepath: V5_read1.fastq.gz (md5: 0d7fafe0640823f2a13723bd7e317ae5)
Quality filter results
Total number of input sequences: 71158
Barcode not in mapping file: 0
Read too short after quality truncation: 2486
Count of N characters exceeds limit: 50
Illumina quality digit = 0: 0
Barcode errors exceed max: 0

Result summary (after quality filtering)

Median sequence length: 301.00
V5 68622

Total number seqs written 68622

Input file paths
Sequence read filepath: V5_read2.fastq.gz (md5: b3f79cee785deae01ed9a54994d93ff2)
Quality filter results
Total number of input sequences: 71158
Barcode not in mapping file: 0
Read too short after quality truncation: 2474
Count of N characters exceeds limit: 72
Illumina quality digit = 0: 0
Barcode errors exceed max: 0

Result summary (after quality filtering)
Median sequence length: 301.00
V5 68612

Total number seqs written 68612

Input file paths
Sequence read filepath: V6_read1.fastq.gz (md5: c58f8670f6f65b47c565b4fe09f67d24)
Quality filter results
Total number of input sequences: 59721
Barcode not in mapping file: 0
Read too short after quality truncation: 1557
Count of N characters exceeds limit: 41
Illumina quality digit = 0: 0
Barcode errors exceed max: 0

Result summary (after quality filtering)
Median sequence length: 301.00
V6 58123

Total number seqs written 58123

Input file paths
Sequence read filepath: V6_read2.fastq.gz (md5: 5439e3d7a694f5f4e6f166c6df1caf94)
Quality filter results

Total number of input sequences: 59721
Barcode not in mapping file: 0
Read too short after quality truncation: 1533
Count of N characters exceeds limit: 60
Illumina quality digit = 0: 0
Barcode errors exceed max: 0

Result summary (after quality filtering)
Median sequence length: 301.00
V6 58128

Total number seqs written 58128

Input file paths
Sequence read filepath: V7_read1.fastq.gz (md5: 21cf12bd75439472cb8f8ebdc10cc923)
Quality filter results
Total number of input sequences: 61455
Barcode not in mapping file: 0
Read too short after quality truncation: 3148
Count of N characters exceeds limit: 71
Illumina quality digit = 0: 0
Barcode errors exceed max: 0

Result summary (after quality filtering)
Median sequence length: 301.00
V7 58236

Total number seqs written 58236

Input file paths
Sequence read filepath: V7_read2.fastq.gz (md5: 2f4b0b7e463fbf924a581a35d695dc41)
Quality filter results
Total number of input sequences: 61455
Barcode not in mapping file: 0
Read too short after quality truncation: 3165
Count of N characters exceeds limit: 66
Illumina quality digit = 0: 0
Barcode errors exceed max: 0

Result summary (after quality filtering)

Median sequence length: 301.00
V7	58224

Total number seqs written	58224

Input file paths
Sequence read filepath: V8_read1.fastq.gz (md5: 3ad6fbe8b07c8459b079b8efc2fefe4d)
Quality filter results
Total number of input sequences: 40521
Barcode not in mapping file: 0
Read too short after quality truncation: 13025
Count of N characters exceeds limit: 28
Illumina quality digit = 0: 0
Barcode errors exceed max: 0

Result summary (after quality filtering)
Median sequence length: 301.00
V8	27468

Total number seqs written	27468

Input file paths
Sequence read filepath: V8_read2.fastq.gz (md5: 00a03d0b6d31f61114ee69634ffb07f3)
Quality filter results
Total number of input sequences: 40521
Barcode not in mapping file: 0
Read too short after quality truncation: 13123
Count of N characters exceeds limit: 33
Illumina quality digit = 0: 0
Barcode errors exceed max: 0

Result summary (after quality filtering)
Median sequence length: 301.00
V8	27365

Total number seqs written	27365

Input file paths
Sequence read filepath: V9_read1.fastq.gz (md5: 22f58a4f014163d62e7cbcde245f0500)
Quality filter results

Total number of input sequences: 52861
Barcode not in mapping file: 0
Read too short after quality truncation: 7228
Count of N characters exceeds limit: 33
Illumina quality digit = 0: 0
Barcode errors exceed max: 0

Result summary (after quality filtering)
Median sequence length: 301.00
V9 45600

Total number seqs written 45600

Input file paths
Sequence read filepath: V9_read2.fastq.gz (md5:
35202a74e3f2b7efdebf374bd3157bb3)
Quality filter results
Total number of input sequences: 52861
Barcode not in mapping file: 0
Read too short after quality truncation: 7231
Count of N characters exceeds limit: 54
Illumina quality digit = 0: 0
Barcode errors exceed max: 0

Result summary (after quality filtering)
Median sequence length: 301.00
V9 45576

Total number seqs written 45576

Input file paths
Sequence read filepath: V10_read1.fastq.gz (md5:
ae1c81e744b20b2ddcd2679bf555856e)
Quality filter results
Total number of input sequences: 25933
Barcode not in mapping file: 0
Read too short after quality truncation: 151
Count of N characters exceeds limit: 51
Illumina quality digit = 0: 0
Barcode errors exceed max: 0

Result summary (after quality filtering)
Median sequence length: 301.00
V10 25731

Total number seqs written 25731

Input file paths
Sequence read filepath: V10_read2.fastq.gz (md5: 53d4bd5ab69e0825f03ff21f08d310a0)
Quality filter results
Total number of input sequences: 25933
Barcode not in mapping file: 0
Read too short after quality truncation: 166
Count of N characters exceeds limit: 23
Illumina quality digit = 0: 0
Barcode errors exceed max: 0

Result summary (after quality filtering)
Median sequence length: 300.00
V10 25744

Total number seqs written 25744

Input file paths
Sequence read filepath: V11_read1.fastq.gz (md5: bd87586f106eb7a264e72c2b312d308d)
Quality filter results
Total number of input sequences: 44167
Barcode not in mapping file: 0
Read too short after quality truncation: 1959
Count of N characters exceeds limit: 32
Illumina quality digit = 0: 0
Barcode errors exceed max: 0

Result summary (after quality filtering)
Median sequence length: 301.00
V11 42176

Total number seqs written 42176

Input file paths
Sequence read filepath: V11_read2.fastq.gz (md5: 74249b1ba7f92ce2cddf753ec693ceb5)
Quality filter results
Total number of input sequences: 44167
Barcode not in mapping file: 0
Read too short after quality truncation: 1945
Count of N characters exceeds limit: 49
Illumina quality digit = 0: 0
Barcode errors exceed max: 0

Result summary (after quality filtering)
Median sequence length: 301.00
V11 42173

Total number seqs written 42173

Input file paths
Sequence read filepath: V12_read1.fastq.gz (md5: ee933bf0a04fc7d837db38efe339a3e3)
Quality filter results
Total number of input sequences: 36355
Barcode not in mapping file: 0
Read too short after quality truncation: 10106
Count of N characters exceeds limit: 23
Illumina quality digit = 0: 0
Barcode errors exceed max: 0

Result summary (after quality filtering)
Median sequence length: 301.00
V12 26226

Total number seqs written 26226

Input file paths
Sequence read filepath: V12_read2.fastq.gz (md5: 926858f867a8d6a999ce8470cee51bd7)
Quality filter results
Total number of input sequences: 36355
Barcode not in mapping file: 0
Read too short after quality truncation: 10275

Count of N characters exceeds limit: 31
Illumina quality digit = 0: 0
Barcode errors exceed max: 0

Result summary (after quality filtering)
Median sequence length: 301.00
V12 26049

Total number seqs written 26049
---

qiime_config values:
pick_otus_reference_seqs_fp
/home/alaricprins/miniconda3/envs/qiime1/lib/python2.7/site-packages/qiime_default_reference/gg_13_8_otus/rep_set/97_otus.fasta
sc_queue all.q
pynast_template_alignment_fp
/home/alaricprins/miniconda3/envs/qiime1/lib/python2.7/site-packages/qiime_default_reference/gg_13_8_otus/rep_set_aligned/85_otus.pynast.fasta
cluster_jobs_fp start_parallel_jobs.py
assign_taxonomy_reference_seqs_fp
/home/alaricprins/miniconda3/envs/qiime1/lib/python2.7/site-packages/qiime_default_reference/gg_13_8_otus/rep_set/97_otus.fasta
torque_queue friendlyq
jobs_to_start 1
denoiser_min_per_core 50
assign_taxonomy_id_to_taxonomy_fp
/home/alaricprins/miniconda3/envs/qiime1/lib/python2.7/site-packages/qiime_default_reference/gg_13_8_otus/taxonomy/97_otu_taxonomy.txt
temp_dir /tmp/
blastall_fp blastall
seconds_to_sleep 1

parameter file values:
parallel:jobs_to_start 4

Input file md5 sums:
slout/seqs.fna: 6e7f1b0f24ad5a160a4e12b8c6dff13f
eztaxon_qiime_full.fasta: f574af1ce44b991a6d30123655f23602

Executing commands.

Pick Reference OTUs command
parallel_pick_otus_uclust_ref.py -i slout/seqs.fna -o otus//step1_otus -r eztaxon_qiime_full.fasta -T --jobs_to_start 4

Stdout:

Stderr:

Generate full failures fasta file command
filter_fasta.py -f slout/seqs.fna -s otus//step1_otus/seqs_failures.txt -o otus//step1_otus/failures.fasta

Stdout:

Stderr:

Executing commands.

Pick rep set command
pick_rep_set.py -i otus//step1_otus/seqs_otus.txt -o otus//step1_otus/step1_rep_set.fna -f slout/seqs.fna

Stdout:

Stderr:

Subsample the failures fasta file using API
python -c "import qiime; qiime.util.subsample_fasta('/home/alaricprins/16S/otus/step1_otus/failures.fasta', '/home/alaricprins/16S/otus/step2_otus/subsampled_failures.fasta', '0.001000')

"Executing commands.

Pick de novo OTUs for new clusters command
pick_otus.py -i otus//step2_otus//subsampled_failures.fasta -o otus//step2_otus/ -m uclust --denovo_otu_id_prefix New.ReferenceOTU

Stdout:

Stderr:

Pick representative set for subsampled failures command

pick_rep_set.py -i otus//step2_otus//subsampled_failures_otus.txt -o otus//step2_otus//step2_rep_set.fna -f otus//step2_otus//subsampled_failures.fasta

Stdout:

Stderr:

Pick reference OTUs using de novo rep set command
parallel_pick_otus_uclust_ref.py -i otus//step1_otus/failures.fasta -o otus//step3_otus/ -r otus//step2_otus//step2_rep_set.fna -T --jobs_to_start 4

Stdout:

Stderr:

Create fasta file of step3 failures command
filter_fasta.py -f otus//step1_otus/failures.fasta -s otus//step3_otus//failures_failures.txt -o otus//step3_otus//failures_failures.fasta

Stdout:

Stderr:

Pick de novo OTUs on step3 failures command
pick_otus.py -i otus//step3_otus//failures_failures.fasta -o otus//step4_otus/ -m uclust --denovo_otu_id_prefix New.CleanUp.ReferenceOTU

Stdout:

Stderr:

Merge OTU maps command
cat otus//step1_otus/seqs_otus.txt otus//step3_otus//failures_otus.txt otus//step4_otus//failures_failures_otus.txt > otus//final_otu_map.txt

Stdout:

Stderr:

Pick representative set for subsampled failures command
pick_rep_set.py -i otus//step4_otus//failures_failures_otus.txt -o otus//step4_otus//step4_rep_set.fna -f otus//step3_otus//failures_failures.fasta

Stdout:

Stderr:

Filter singletons from the otu map using API
python -c "import qiime;
qiime.filter.filter_otus_from_otu_map('/home/alaricprins/16S/otus/final_otu_map.txt'
, '/home/alaricprins/16S/otus/final_otu_map_mc2.txt', '2')"

Write non-singleton otus representative sequences from step1 to the final rep set
file: otus//rep_set.fna

Copy the full input refseqs file to the new refseq file
cp eztaxon_qiime_full.fasta otus//new_refseqs.fna

Write non-singleton otus representative sequences from step 2 and step 4 to the
final representative set and the new reference set (otus//rep_set.fna and
otus//new_refseqs.fna respectively)

Executing commands.

Make the otu table command
make_otu_table.py -i otus//final_otu_map_mc2.txt -o otus//otu_table_mc2.biom

Stdout:

Stderr:

Executing commands.

Assign taxonomy command
parallel_assign_taxonomy_uclust.py -i otus//rep_set.fna -o
otus//uclust_assigned_taxonomy -T --jobs_to_start 4

Stdout:

Stderr:

Executing commands.

Add taxa to OTU table command
biom add-metadata -i otus//otu_table_mc2.biom --observation-metadata-fp
otus//uclust_assigned_taxonomy/rep_set_tax_assignments.txt -o

otus//otu_table_mc2_w_tax.biom --sc-separated taxonomy --observation-header OTUID,taxonomy

Stdout:

Stderr:

Executing commands.

Align sequences command
parallel_align_seqs_pynast.py -i otus//rep_set.fna -o otus//pynast_aligned_seqs -T --jobs_to_start 4

Stdout:

Stderr:

Filter alignment command
filter_alignment.py -o otus//pynast_aligned_seqs -i otus//pynast_aligned_seqs/rep_set_aligned.fasta

Stdout:

Stderr:

Build phylogenetic tree command
make_phylogeny.py -i otus//pynast_aligned_seqs/rep_set_aligned_pfiltered.fasta -o otus//rep_set.tre

Stdout:

Stderr:

Executing commands.

qiime_config values:
pick_otus_reference_seqs_fp
/home/alaricprins/miniconda3/envs/qiime1/lib/python2.7/site-packages/qiime_default_reference/gg_13_8_otus/rep_set/97_otus.fasta
sc_queue all.q
pynast_template_alignment_fp
/home/alaricprins/miniconda3/envs/qiime1/lib/python2.7/site-

packages/qiime_default_reference/gg_13_8_otus/rep_set_aligned/85_otus.pynast.fast
a
cluster_jobs_fp	start_parallel_jobs.py
assign_taxonomy_reference_seqs_fp
	/home/alaricprins/miniconda3/envs/qiime1/lib/python2.7/site-
packages/qiime_default_reference/gg_13_8_otus/rep_set/97_otus.fasta
torque_queue	friendlyq
jobs_to_start	1
denoiser_min_per_core	50
assign_taxonomy_id_to_taxonomy_fp
	/home/alaricprins/miniconda3/envs/qiime1/lib/python2.7/site-
packages/qiime_default_reference/gg_13_8_otus/taxonomy/97_otu_taxonomy.txt
temp_dir	/tmp/
blastall_fp	blastall
seconds_to_sleep	1

parameter file values:
parallel:jobs_to_start	1

Input file md5 sums:
otus/otu_table_mc2_w_tax_peat_16s.biom: d3810b244035a764c05825f6cd8fee2e
map_16S.txt: 717103108b9eee2684fc8a5212f246a1
otus/rep_set.tre: 2bb1d3a6bc88d40db71034c39b730730

Skipping 'biom summarize-table' as cdout//biom_table_summary.txt exists.

Executing commands.

Filter low sequence count samples from table (minimum sequence count: 1060)
command
filter_samples_from_otu_table.py -i otus/otu_table_mc2_w_tax_peat_16s.biom -o
cdout//table_mc1060.biom -n 1060

Stdout:

Stderr:

Rarify the OTU table to 1060 sequences/sample command
single_rarefaction.py -i cdout//table_mc1060.biom -o cdout//table_even1060.biom -d
1060

Stdout:

Stderr:

Skipping beta_diversity_through_plots.py as cdout//bdiv_even1060/weighted_unifrac_dm.txt, cdout//bdiv_even1060/unweighted_unifrac_dm.txt exist(s).

Skipping alpha_rarefaction.py as cdout//arare_max1060//alpha_rarefaction_plots/rarefaction_plots.html exists.

Skipping summarize_taxa_through_plots.py for as cdout//taxa_plots/taxa_summary_plots/area_charts.html, cdout//taxa_plots/taxa_summary_plots/bar_charts.html exist(s).

Executing commands.

Collapse samples in OTU table by categories command
collapse_samples.py -m map_16S.txt -b cdout//table_mc1060.biom --output_biom_fp cdout//taxa_plots_Site/Site_otu_table.biom --output_mapping_fp cdout//taxa_plots_Site/Site_map.txt --collapse_fields 'Site'

Stdout:

Stderr:

Sort OTU Table command
sort_otu_table.py -i cdout//taxa_plots_Site/Site_otu_table.biom -o cdout//taxa_plots_Site/Site_otu_table_sorted.biom

Stdout:

Stderr:

Summarize Taxonomy command
summarize_taxa.py -i cdout//taxa_plots_Site/Site_otu_table_sorted.biom -o cdout//taxa_plots_Site/

Stdout:

Stderr:

Plot Taxonomy Summary command
plot_taxa_summary.py -i
cdout//taxa_plots_Site/Site_otu_table_sorted_L2.txt,cdout//taxa_plots_Site/Site_otu_t

able_sorted_L3.txt,cdout//taxa_plots_Site/Site_otu_table_sorted_L4.txt,cdout//taxa_plots_Site/Site_otu_table_sorted_L5.txt,cdout//taxa_plots_Site/Site_otu_table_sorted_L6.txt -o cdout//taxa_plots_Site//taxa_summary_plots/

Stdout:

Stderr:
/home/alaricprins/miniconda3/envs/qiime1/lib/python2.7/site-packages/matplotlib/collections.py:590: FutureWarning: elementwise comparison failed; returning scalar instead, but in the future will perform elementwise comparison
  if self._edgecolors == str('face'):

Executing commands.

Collapse samples in OTU table by categories command
collapse_samples.py -m map_16S.txt -b cdout//table_mc1060.biom --output_biom_fp cdout//taxa_plots_Sample/Sample_otu_table.biom --output_mapping_fp cdout//taxa_plots_Sample/Sample_map.txt --collapse_fields 'Sample'

Stdout:

Stderr:

Sort OTU Table command
sort_otu_table.py -i cdout//taxa_plots_Sample/Sample_otu_table.biom -o cdout//taxa_plots_Sample/Sample_otu_table_sorted.biom

Stdout:

Stderr:

Summarize Taxonomy command
summarize_taxa.py -i cdout//taxa_plots_Sample/Sample_otu_table_sorted.biom -o cdout//taxa_plots_Sample/

Stdout:

Stderr:

Plot Taxonomy Summary command
plot_taxa_summary.py -i
cdout//taxa_plots_Sample/Sample_otu_table_sorted_L2.txt,cdout//taxa_plots_Sampl

e/Sample_otu_table_sorted_L3.txt,cdout//taxa_plots_Sample/Sample_otu_table_sorted_L4.txt,cdout//taxa_plots_Sample/Sample_otu_table_sorted_L5.txt,cdout//taxa_plots_Sample/Sample_otu_table_sorted_L6.txt -o cdout//taxa_plots_Sample//taxa_summary_plots/

Stdout:

Stderr:
/home/alaricprins/miniconda3/envs/qiime1/lib/python2.7/site-packages/matplotlib/collections.py:590: FutureWarning: elementwise comparison failed; returning scalar instead, but in the future will perform elementwise comparison
  if self._edgecolors == str('face'):

Executing commands.

Collapse samples in OTU table by categories command
collapse_samples.py -m map_16S.txt -b cdout//table_mc1060.biom --output_biom_fp cdout//taxa_plots_Description/Description_otu_table.biom --output_mapping_fp cdout//taxa_plots_Description/Description_map.txt --collapse_fields 'Description'

Stdout:

Stderr:

Sort OTU Table command
sort_otu_table.py -i cdout//taxa_plots_Description/Description_otu_table.biom -o cdout//taxa_plots_Description/Description_otu_table_sorted.biom

Stdout:

Stderr:

Summarize Taxonomy command
summarize_taxa.py -i cdout//taxa_plots_Description/Description_otu_table_sorted.biom -o cdout//taxa_plots_Description/

Stdout:

Stderr:

Plot Taxonomy Summary command

plot_taxa_summary.py -i
cdout//taxa_plots_Description/Description_otu_table_sorted_L2.txt,cdout//taxa_plot
s_Description/Description_otu_table_sorted_L3.txt,cdout//taxa_plots_Description/D
escription_otu_table_sorted_L4.txt,cdout//taxa_plots_Description/Description_otu_t
able_sorted_L5.txt,cdout//taxa_plots_Description/Description_otu_table_sorted_L6.t
xt -o cdout//taxa_plots_Description//taxa_summary_plots/

Stdout:

Stderr:
/home/alaricprins/miniconda3/envs/qiime1/lib/python2.7/site-
packages/matplotlib/collections.py:590: FutureWarning: elementwise comparison
failed; returning scalar instead, but in the future will perform elementwise
comparison
  if self._edgecolors == str('face'):

Skipping compressing of filtered BIOM table as cdout//table_mc1060.biom.gz exists.

Skipping compressing of rarefied BIOM table as cdout//table_even1060.biom.gz
exists.

Executing commands.

Boxplots (Site) command
make_distance_boxplots.py -d cdout//bdiv_even1060/weighted_unifrac_dm.txt -f
Site -o cdout//bdiv_even1060//weighted_unifrac_boxplots/ -m map_16S.txt -n 999

Stdout:

Stderr:

Boxplots (Sample) command
make_distance_boxplots.py -d cdout//bdiv_even1060/weighted_unifrac_dm.txt -f
Sample -o cdout//bdiv_even1060//weighted_unifrac_boxplots/ -m map_16S.txt -n 999

Stdout:

Stderr:

Boxplots (Description) command
make_distance_boxplots.py -d cdout//bdiv_even1060/weighted_unifrac_dm.txt -f
Description -o cdout//bdiv_even1060//weighted_unifrac_boxplots/ -m map_16S.txt -
n 999

Stdout:

Stderr:

Boxplots (Site) command
make_distance_boxplots.py -d cdout//bdiv_even1060/unweighted_unifrac_dm.txt -f Site -o cdout//bdiv_even1060//unweighted_unifrac_boxplots/ -m map_16S.txt -n 999

Stdout:

Stderr:

Boxplots (Sample) command
make_distance_boxplots.py -d cdout//bdiv_even1060/unweighted_unifrac_dm.txt -f Sample -o cdout//bdiv_even1060//unweighted_unifrac_boxplots/ -m map_16S.txt -n 999

Stdout:

Stderr:

Boxplots (Description) command
make_distance_boxplots.py -d cdout//bdiv_even1060/unweighted_unifrac_dm.txt -f Description -o cdout//bdiv_even1060//unweighted_unifrac_boxplots/ -m map_16S.txt -n 999

Stdout:

Stderr:

Compare alpha diversity (chao1) command
compare_alpha_diversity.py -i cdout//arare_max1060//alpha_div_collated/chao1.txt -m map_16S.txt -c Site,Sample,Description -o cdout//arare_max1060//compare_chao1 -n 999

Stdout:

Stderr:

Compare alpha diversity (PD_whole_tree) command

compare_alpha_diversity.py -i cdout//arare_max1060//alpha_div_collated/PD_whole_tree.txt -m map_16S.txt -c Site,Sample,Description -o cdout//arare_max1060//compare_PD_whole_tree -n 999

Stdout:

Stderr:

Compare alpha diversity (observed_otus) command
compare_alpha_diversity.py -i cdout//arare_max1060//alpha_div_collated/observed_otus.txt -m map_16S.txt -c Site,Sample,Description -o cdout//arare_max1060//compare_observed_otus -n 999

Stdout:

Stderr:

Group significance (Site) command
group_significance.py -i cdout//table_even1060.biom -m map_16S.txt -c Site -o cdout//group_significance_Site.txt

Stdout:

Stderr:

Group significance (Sample) command
group_significance.py -i cdout//table_even1060.biom -m map_16S.txt -c Sample -o cdout//group_significance_Sample.txt

Stdout:

Stderr:

Group significance (Description) command
group_significance.py -i cdout//table_even1060.biom -m map_16S.txt -c Description -o cdout//group_significance_Description.txt

Stdout:

Stderr:

Logging stopped at 17:31:25 on 18 Aug 2017

QIIME analyses log - ITS

Input file paths
Sequence read filepath: 1A_read1.fastq.gz (md5: 642513ea498e5bce2431bd70e7093a97)
Quality filter results
Total number of input sequences: 12421
Barcode not in mapping file: 0
Read too short after quality truncation: 902
Count of N characters exceeds limit: 0
Illumina quality digit = 0: 0
Barcode errors exceed max: 0

Result summary (after quality filtering)
Median sequence length: 301.00
1A	11519

Total number seqs written 11519

Input file paths
Sequence read filepath: 1A_read2.fastq.gz (md5: 6ba023db01ca264ed582a4f8e4628cdd)
Quality filter results
Total number of input sequences: 12421
Barcode not in mapping file: 0
Read too short after quality truncation: 830
Count of N characters exceeds limit: 0
Illumina quality digit = 0: 0
Barcode errors exceed max: 0

Result summary (after quality filtering)
Median sequence length: 301.00
1A	11591

Total number seqs written 11591

Input file paths
Sequence read filepath: 1B_read1.fastq.gz (md5: e06d5f8b298a15624363f367eb5c4767)
Quality filter results
Total number of input sequences: 4969
Barcode not in mapping file: 0

Read too short after quality truncation: 299
Count of N characters exceeds limit: 0
Illumina quality digit = 0: 0
Barcode errors exceed max: 0

Result summary (after quality filtering)
Median sequence length: 301.00
1B 4670

Total number seqs written 4670

Input file paths
Sequence read filepath: 1B_read2.fastq.gz (md5: 827187735f4cd5185b461d7d62123549)
Quality filter results
Total number of input sequences: 4969
Barcode not in mapping file: 0
Read too short after quality truncation: 287
Count of N characters exceeds limit: 0
Illumina quality digit = 0: 0
Barcode errors exceed max: 0

Result summary (after quality filtering)
Median sequence length: 301.00
1B 4682

Total number seqs written 4682

Input file paths
Sequence read filepath: 1C_read1.fastq.gz (md5: 36ff58da5d93b27e75f472ba8fdc1988)
Quality filter results
Total number of input sequences: 7132
Barcode not in mapping file: 0
Read too short after quality truncation: 563
Count of N characters exceeds limit: 0
Illumina quality digit = 0: 0
Barcode errors exceed max: 0

Result summary (after quality filtering)
Median sequence length: 301.00
1C 6569

Total number seqs written 6569

Input file paths
Sequence read filepath: 1C_read2.fastq.gz (md5: 491c978c4cedac1913a5aeabdd2992d8)
Quality filter results
Total number of input sequences: 7132
Barcode not in mapping file: 0
Read too short after quality truncation: 523
Count of N characters exceeds limit: 0
Illumina quality digit = 0: 0
Barcode errors exceed max: 0

Result summary (after quality filtering)
Median sequence length: 301.00
1C 6609

Total number seqs written 6609

Input file paths
Sequence read filepath: 2A_read1.fastq.gz (md5: a5efedecff1bc80cf77500b6127d14e5)
Quality filter results
Total number of input sequences: 136265
Barcode not in mapping file: 0
Read too short after quality truncation: 14143
Count of N characters exceeds limit: 69
Illumina quality digit = 0: 0
Barcode errors exceed max: 0

Result summary (after quality filtering)
Median sequence length: 301.00
2A 122053

Total number seqs written 122053

Input file paths
Sequence read filepath: 2A_read2.fastq.gz (md5: 2868c3fc81464a5c033fc74d3d8602ab)
Quality filter results

Total number of input sequences: 136265
Barcode not in mapping file: 0
Read too short after quality truncation: 13622
Count of N characters exceeds limit: 115
Illumina quality digit = 0: 0
Barcode errors exceed max: 0

Result summary (after quality filtering)
Median sequence length: 301.00
2A	122528

Total number seqs written	122528

Input file paths
Sequence read filepath: 2B_read1.fastq.gz (md5: 34f34b2be43c5bba1ce4ec16b182a5d7)
Quality filter results
Total number of input sequences: 9959
Barcode not in mapping file: 0
Read too short after quality truncation: 1546
Count of N characters exceeds limit: 0
Illumina quality digit = 0: 0
Barcode errors exceed max: 0

Result summary (after quality filtering)
Median sequence length: 301.00
2B	8413

Total number seqs written	8413

Input file paths
Sequence read filepath: 2B_read2.fastq.gz (md5: c1283d51c375d598f39be8ac58a79daf)
Quality filter results
Total number of input sequences: 9959
Barcode not in mapping file: 0
Read too short after quality truncation: 1411
Count of N characters exceeds limit: 0
Illumina quality digit = 0: 0
Barcode errors exceed max: 0

Result summary (after quality filtering)

Median sequence length: 301.00
2B 8548

Total number seqs written 8548

Input file paths
Sequence read filepath: 2C_read1.fastq.gz (md5: 5f37d50c08ac20c8c4aaaa02937dcf28)
Quality filter results
Total number of input sequences: 9068
Barcode not in mapping file: 0
Read too short after quality truncation: 152
Count of N characters exceeds limit: 0
Illumina quality digit = 0: 0
Barcode errors exceed max: 0

Result summary (after quality filtering)
Median sequence length: 301.00
2C 8916

Total number seqs written 8916

Input file paths
Sequence read filepath: 2C_read2.fastq.gz (md5: 68b3b53e08549374470ec462b0f0bd2d)
Quality filter results
Total number of input sequences: 9068
Barcode not in mapping file: 0
Read too short after quality truncation: 685
Count of N characters exceeds limit: 0
Illumina quality digit = 0: 0
Barcode errors exceed max: 0

Result summary (after quality filtering)
Median sequence length: 301.00
2C 8383

Total number seqs written 8383

Input file paths

Sequence read filepath: G1_read1.fastq.gz (md5: be640732c8f0d796228c671682b0bd62)
Quality filter results
Total number of input sequences: 80048
Barcode not in mapping file: 0
Read too short after quality truncation: 1783
Count of N characters exceeds limit: 73
Illumina quality digit = 0: 0
Barcode errors exceed max: 0

Result summary (after quality filtering)
Median sequence length: 301.00
G1 78192

Total number seqs written 78192

Input file paths
Sequence read filepath: G1_read2.fastq.gz (md5: 3ab3c3011509fa476876eb38b7411289)
Quality filter results
Total number of input sequences: 80048
Barcode not in mapping file: 0
Read too short after quality truncation: 1768
Count of N characters exceeds limit: 81
Illumina quality digit = 0: 0
Barcode errors exceed max: 0

Result summary (after quality filtering)
Median sequence length: 301.00
G1 78199

Total number seqs written 78199

Input file paths
Sequence read filepath: G2_read1.fastq.gz (md5: 35d7f3d381b8e7e082a216b17d9d2f0e)
Quality filter results
Total number of input sequences: 621
Barcode not in mapping file: 0
Read too short after quality truncation: 133
Count of N characters exceeds limit: 0

Illumina quality digit = 0: 0
Barcode errors exceed max: 0

Result summary (after quality filtering)
Median sequence length: 301.00
G2 488

Total number seqs written 488

Input file paths
Sequence read filepath: G2_read2.fastq.gz (md5: 294c50a80c9c79c3222c6a4890203509)
Quality filter results
Total number of input sequences: 621
Barcode not in mapping file: 0
Read too short after quality truncation: 128
Count of N characters exceeds limit: 0
Illumina quality digit = 0: 0
Barcode errors exceed max: 0

Result summary (after quality filtering)
Median sequence length: 301.00
G2 493

Total number seqs written 493

Input file paths
Sequence read filepath: G3_read1.fastq.gz (md5: 6e4cc3d0ebe22e0f6a55378f126396dc)
Quality filter results
Total number of input sequences: 86172
Barcode not in mapping file: 0
Read too short after quality truncation: 1235
Count of N characters exceeds limit: 64
Illumina quality digit = 0: 0
Barcode errors exceed max: 0

Result summary (after quality filtering)
Median sequence length: 301.00
G3 84873

Total number seqs written 84873

Input file paths
Sequence read filepath: G3_read2.fastq.gz (md5: 414b1987893fa70afc7843554dbfbf2e)
Quality filter results
Total number of input sequences: 86172
Barcode not in mapping file: 0
Read too short after quality truncation: 1245
Count of N characters exceeds limit: 90
Illumina quality digit = 0: 0
Barcode errors exceed max: 0

Result summary (after quality filtering)
Median sequence length: 301.00
G3 84837

Total number seqs written 84837

Input file paths
Sequence read filepath: G4_read1.fastq.gz (md5:
de677300b527db16d9a6c1f63d7f49e6)
Quality filter results
Total number of input sequences: 85469
Barcode not in mapping file: 0
Read too short after quality truncation: 1018
Count of N characters exceeds limit: 77
Illumina quality digit = 0: 0
Barcode errors exceed max: 0

Result summary (after quality filtering)
Median sequence length: 301.00
G4 84374

Total number seqs written 84374

Input file paths
Sequence read filepath: G4_read2.fastq.gz (md5: dee50e4c901405866c08a2cef80297df)
Quality filter results
Total number of input sequences: 85469
Barcode not in mapping file: 0
Read too short after quality truncation: 1003

Count of N characters exceeds limit: 88
Illumina quality digit = 0: 0
Barcode errors exceed max: 0

Result summary (after quality filtering)
Median sequence length: 301.00
G4 84378

Total number seqs written 84378

Input file paths
Sequence read filepath: G5_read1.fastq.gz (md5: 2c378addd102f65810a32035b7e77d8c)
Quality filter results
Total number of input sequences: 2225
Barcode not in mapping file: 0
Read too short after quality truncation: 380
Count of N characters exceeds limit: 0
Illumina quality digit = 0: 0
Barcode errors exceed max: 0

Result summary (after quality filtering)
Median sequence length: 301.00
G5 1845

Total number seqs written 1845

Input file paths
Sequence read filepath: G5_read2.fastq.gz (md5: 61d93c91e6e2c82d0a2c2f7f823ee711)
Quality filter results
Total number of input sequences: 2225
Barcode not in mapping file: 0
Read too short after quality truncation: 359
Count of N characters exceeds limit: 0
Illumina quality digit = 0: 0
Barcode errors exceed max: 0

Result summary (after quality filtering)
Median sequence length: 301.00
G5 1866

Total number seqs written 1866

Input file paths
Sequence read filepath: G6_read1.fastq.gz (md5: ed1660dec1a8125e288d3ed036023d66)
Quality filter results
Total number of input sequences: 2091
Barcode not in mapping file: 0
Read too short after quality truncation: 158
Count of N characters exceeds limit: 1
Illumina quality digit = 0: 0
Barcode errors exceed max: 0

Result summary (after quality filtering)
Median sequence length: 301.00
G6 1932

Total number seqs written 1932

Input file paths
Sequence read filepath: G6_read2.fastq.gz (md5: 0253ee4aff7788d51c8b7951d433aa5f)
Quality filter results
Total number of input sequences: 2091
Barcode not in mapping file: 0
Read too short after quality truncation: 171
Count of N characters exceeds limit: 0
Illumina quality digit = 0: 0
Barcode errors exceed max: 0

Result summary (after quality filtering)
Median sequence length: 300.00
G6 1920

Total number seqs written 1920

Input file paths
Sequence read filepath: G7_read1.fastq.gz (md5: ef2e3089b3592ce185be01b2fac72cf5)
Quality filter results
Total number of input sequences: 41049
Barcode not in mapping file: 0

Read too short after quality truncation: 3938
Count of N characters exceeds limit: 29
Illumina quality digit = 0: 0
Barcode errors exceed max: 0

Result summary (after quality filtering)
Median sequence length: 301.00
G7 37082

Total number seqs written 37082

Input file paths
Sequence read filepath: G7_read2.fastq.gz (md5: 1c2417ab0d97f1f9aa718a33132e43d0)
Quality filter results
Total number of input sequences: 41049
Barcode not in mapping file: 0
Read too short after quality truncation: 3709
Count of N characters exceeds limit: 40
Illumina quality digit = 0: 0
Barcode errors exceed max: 0

Result summary (after quality filtering)
Median sequence length: 301.00
G7 37300

Total number seqs written 37300

Input file paths
Sequence read filepath: G8_read1.fastq.gz (md5: 43f38193450a9a41a842ec1f504a3fe3)
Quality filter results
Total number of input sequences: 685
Barcode not in mapping file: 0
Read too short after quality truncation: 143
Count of N characters exceeds limit: 0
Illumina quality digit = 0: 0
Barcode errors exceed max: 0

Result summary (after quality filtering)
Median sequence length: 301.00
G8 542

Total number seqs written 542

Input file paths
Sequence read filepath: G8_read2.fastq.gz (md5: 92c2d1b32d5c93ca2a56ef0c135e52dd)
Quality filter results
Total number of input sequences: 685
Barcode not in mapping file: 0
Read too short after quality truncation: 137
Count of N characters exceeds limit: 0
Illumina quality digit = 0: 0
Barcode errors exceed max: 0

Result summary (after quality filtering)
Median sequence length: 301.00
G8 548

Total number seqs written 548

Input file paths
Sequence read filepath: G9_read1.fastq.gz (md5: 9454018cf9415afb57d5180260b20759)
Quality filter results
Total number of input sequences: 70404
Barcode not in mapping file: 0
Read too short after quality truncation: 1558
Count of N characters exceeds limit: 56
Illumina quality digit = 0: 0
Barcode errors exceed max: 0

Result summary (after quality filtering)
Median sequence length: 301.00
G9 68790

Total number seqs written 68790

Input file paths
Sequence read filepath: G9_read2.fastq.gz (md5: a79a720e77beca0c31d8a72697377f75)

Quality filter results
Total number of input sequences: 70404
Barcode not in mapping file: 0
Read too short after quality truncation: 1546
Count of N characters exceeds limit: 65
Illumina quality digit = 0: 0
Barcode errors exceed max: 0

Result summary (after quality filtering)
Median sequence length: 301.00
G9 68793

Total number seqs written 68793

Input file paths
Sequence read filepath: V1_read1.fastq.gz (md5: 786062dcab125fe9a7bc9e61f29bec9e)
Quality filter results
Total number of input sequences: 28159
Barcode not in mapping file: 0
Read too short after quality truncation: 83
Count of N characters exceeds limit: 51
Illumina quality digit = 0: 0
Barcode errors exceed max: 0

Result summary (after quality filtering)
Median sequence length: 301.00
V1 28025

Total number seqs written 28025

Input file paths
Sequence read filepath: V1_read2.fastq.gz (md5: c26e2480b6ff747a1dc85006af9069ea)
Quality filter results
Total number of input sequences: 28159
Barcode not in mapping file: 0
Read too short after quality truncation: 95
Count of N characters exceeds limit: 32
Illumina quality digit = 0: 0
Barcode errors exceed max: 0

Result summary (after quality filtering)

Median sequence length: 300.00
V1 28032

Total number seqs written 28032

Input file paths
Sequence read filepath: V2_read1.fastq.gz (md5: a05102513eb8d71a494294fb8d96efb9)
Quality filter results
Total number of input sequences: 34176
Barcode not in mapping file: 0
Read too short after quality truncation: 326
Count of N characters exceeds limit: 72
Illumina quality digit = 0: 0
Barcode errors exceed max: 0

Result summary (after quality filtering)
Median sequence length: 300.00
V2 33778

Total number seqs written 33778

Input file paths
Sequence read filepath: V2_read2.fastq.gz (md5: 965a22e0e394871ee2adcc8d3d788eb5)
Quality filter results
Total number of input sequences: 34176
Barcode not in mapping file: 0
Read too short after quality truncation: 323
Count of N characters exceeds limit: 36
Illumina quality digit = 0: 0
Barcode errors exceed max: 0

Result summary (after quality filtering)
Median sequence length: 300.00
V2 33817

Total number seqs written 33817

Input file paths

Sequence read filepath: V3_read1.fastq.gz (md5: d32435b8494cd35c511ddee29e593ae5)
Quality filter results
Total number of input sequences: 2680
Barcode not in mapping file: 0
Read too short after quality truncation: 110
Count of N characters exceeds limit: 0
Illumina quality digit = 0: 0
Barcode errors exceed max: 0

Result summary (after quality filtering)
Median sequence length: 301.00
V3 2570

Total number seqs written 2570

Input file paths
Sequence read filepath: V3_read2.fastq.gz (md5: 63e3d816a622cf0d23a263ab0e331d6d)
Quality filter results
Total number of input sequences: 2680
Barcode not in mapping file: 0
Read too short after quality truncation: 116
Count of N characters exceeds limit: 0
Illumina quality digit = 0: 0
Barcode errors exceed max: 0

Result summary (after quality filtering)
Median sequence length: 301.00
V3 2564

Total number seqs written 2564

Input file paths
Sequence read filepath: V5_read1.fastq.gz (md5: 095008b6327cd393fb9d11637572f436)
Quality filter results
Total number of input sequences: 5669
Barcode not in mapping file: 0
Read too short after quality truncation: 349
Count of N characters exceeds limit: 0

Illumina quality digit = 0: 0
Barcode errors exceed max: 0

Result summary (after quality filtering)
Median sequence length: 301.00
V5 5320

Total number seqs written 5320

Input file paths
Sequence read filepath: V5_read2.fastq.gz (md5: 6badc2cc451f83ca82aabf8260a332fa)
Quality filter results
Total number of input sequences: 5669
Barcode not in mapping file: 0
Read too short after quality truncation: 338
Count of N characters exceeds limit: 0
Illumina quality digit = 0: 0
Barcode errors exceed max: 0

Result summary (after quality filtering)
Median sequence length: 301.00
V5 5331

Total number seqs written 5331

Input file paths
Sequence read filepath: V6_read1.fastq.gz (md5: c8e0c4b6817902fecb0cec3bef0c76f3)
Quality filter results
Total number of input sequences: 66521
Barcode not in mapping file: 0
Read too short after quality truncation: 9258
Count of N characters exceeds limit: 38
Illumina quality digit = 0: 0
Barcode errors exceed max: 0

Result summary (after quality filtering)
Median sequence length: 301.00
V6 57225

Total number seqs written 57225

Input file paths
Sequence read filepath: V6_read2.fastq.gz (md5: fc9709f5017f46a359d5030d7030b3d4)
Quality filter results
Total number of input sequences: 66521
Barcode not in mapping file: 0
Read too short after quality truncation: 9310
Count of N characters exceeds limit: 57
Illumina quality digit = 0: 0
Barcode errors exceed max: 0

Result summary (after quality filtering)
Median sequence length: 301.00
V6 57154

Total number seqs written 57154

Input file paths
Sequence read filepath: V7_read1.fastq.gz (md5: 4b3f4ead15ab51d811a481604894608a)
Quality filter results
Total number of input sequences: 77662
Barcode not in mapping file: 0
Read too short after quality truncation: 36198
Count of N characters exceeds limit: 25
Illumina quality digit = 0: 0
Barcode errors exceed max: 0

Result summary (after quality filtering)
Median sequence length: 300.00
V7 41439

Total number seqs written 41439

Input file paths
Sequence read filepath: V7_read2.fastq.gz (md5: d0078bdd730ee3036abdb00bc028167b)
Quality filter results
Total number of input sequences: 77662
Barcode not in mapping file: 0

Read too short after quality truncation: 36043
Count of N characters exceeds limit: 51
Illumina quality digit = 0: 0
Barcode errors exceed max: 0

Result summary (after quality filtering)
Median sequence length: 300.00
V7 41568

Total number seqs written 41568

Input file paths
Sequence read filepath: V9_read1.fastq.gz (md5: 410ddf1b69f30149b304c9710d5a9c62)
Quality filter results
Total number of input sequences: 72267
Barcode not in mapping file: 0
Read too short after quality truncation: 14384
Count of N characters exceeds limit: 40
Illumina quality digit = 0: 0
Barcode errors exceed max: 0

Result summary (after quality filtering)
Median sequence length: 176.00
V9 57843

Total number seqs written 57843

Input file paths
Sequence read filepath: V9_read2.fastq.gz (md5: 23b5b5ebc84a5fcc713e5e8f08a19de2)
Quality filter results
Total number of input sequences: 72267
Barcode not in mapping file: 0
Read too short after quality truncation: 43170
Count of N characters exceeds limit: 27
Illumina quality digit = 0: 0
Barcode errors exceed max: 0

Result summary (after quality filtering)
Median sequence length: 300.00
V9 29070

Total number seqs written 29070

Input file paths
Sequence read filepath: V10_read1.fastq.gz (md5: cf3eae1a91468a99cff7c0e5e7265f3d)
Quality filter results
Total number of input sequences: 10541
Barcode not in mapping file: 0
Read too short after quality truncation: 25
Count of N characters exceeds limit: 22
Illumina quality digit = 0: 0
Barcode errors exceed max: 0

Result summary (after quality filtering)
Median sequence length: 301.00
V10 10494

Total number seqs written 10494

Input file paths
Sequence read filepath: V10_read2.fastq.gz (md5:
03ebca1fae89b6dec767bd9ad73e08d2)
Quality filter results
Total number of input sequences: 10541
Barcode not in mapping file: 0
Read too short after quality truncation: 26
Count of N characters exceeds limit: 19
Illumina quality digit = 0: 0
Barcode errors exceed max: 0

Result summary (after quality filtering)
Median sequence length: 300.00
V10 10496

Total number seqs written 10496

Input file paths
Sequence read filepath: V11_read1.fastq.gz (md5:
abc995ca4fd81dc3dce2c22cade8d5f4)
Quality filter results

Total number of input sequences: 158694
Barcode not in mapping file: 0
Read too short after quality truncation: 15957
Count of N characters exceeds limit: 113
Illumina quality digit = 0: 0
Barcode errors exceed max: 0

Result summary (after quality filtering)
Median sequence length: 300.00
V11 142624

Total number seqs written 142624

Input file paths
Sequence read filepath: V11_read2.fastq.gz (md5:
720ef1696bbd5970cd0a7d0733c362cc)
Quality filter results
Total number of input sequences: 158694
Barcode not in mapping file: 0
Read too short after quality truncation: 15800
Count of N characters exceeds limit: 132
Illumina quality digit = 0: 0
Barcode errors exceed max: 0

Result summary (after quality filtering)
Median sequence length: 301.00
V11 142762

Total number seqs written 142762
---

qiime_config values:
pick_otus_reference_seqs_fp
/Users/alaricprins/miniconda3/envs/qiime1/lib/python2.7/site-packages/qiime_default_reference/gg_13_8_otus/rep_set/97_otus.fasta
sc_queue all.q
pynast_template_alignment_fp
/Users/alaricprins/miniconda3/envs/qiime1/lib/python2.7/site-packages/qiime_default_reference/gg_13_8_otus/rep_set_aligned/85_otus.pynast.fasta
cluster_jobs_fp start_parallel_jobs.py

assign_taxonomy_reference_seqs_fp
/Users/alaricprins/miniconda3/envs/qiime1/lib/python2.7/site-packages/qiime_default_reference/gg_13_8_otus/rep_set/97_otus.fasta
torque_queue friendlyq
jobs_to_start 1
denoiser_min_per_core 50
assign_taxonomy_id_to_taxonomy_fp
/Users/alaricprins/miniconda3/envs/qiime1/lib/python2.7/site-packages/qiime_default_reference/gg_13_8_otus/taxonomy/97_otu_taxonomy.txt
temp_dir /var/folders/fp/n478ts1n2ps67vp0g30046tr0000gn/T/
blastall_fp blastall
seconds_to_sleep 1

parameter file values:
assign_taxonomy:reference_seqs_fp its_12_11_otus/rep_set/97_otus.fasta
assign_taxonomy:id_to_taxonomy_fp
its_12_11_otus/taxonomy/97_otu_taxonomy.txt
assign_taxonomy:assignment_method blast
parallel:jobs_to_start 1
pick_otus:enable_rev_strand_match True

Input file md5 sums:
slout/seqs.fna: 857b0cec48aecf02df63bab57e288f86
its_12_11_otus/rep_set/97_otus.fasta: 43064e49574c847df9e03acfc7115e02

Forcing --suppress_new_clusters as this is reference-based OTU picking.

Executing commands.

Pick Reference OTUs command
pick_otus.py -i slout/seqs.fna -o otus//step1_otus -r its_12_11_otus/rep_set/97_otus.fasta -m uclust_ref --enable_rev_strand_match --suppress_new_clusters

Stdout:

Stderr:

Generate full failures fasta file command
filter_fasta.py -f slout/seqs.fna -s otus//step1_otus/seqs_failures.txt -o otus//step1_otus/failures.fasta

Stdout:

Stderr:

Executing commands.

Pick rep set command
pick_rep_set.py -i otus//step1_otus/seqs_otus.txt -o otus//step1_otus/step1_rep_set.fna -f slout/seqs.fna

Stdout:

Stderr:

Subsample the failures fasta file using API
python -c "import qiime;
qiime.util.subsample_fasta('/Users/alaricprins/ITS/otus/step1_otus/failures.fasta', '/Users/alaricprins/ITS/otus/step2_otus/subsampled_failures.fasta', '0.001000')

"Forcing --suppress_new_clusters as this is reference-based OTU picking.

Executing commands.

Pick de novo OTUs for new clusters command
pick_otus.py -i otus//step2_otus//subsampled_failures.fasta -o otus//step2_otus/ -m uclust --denovo_otu_id_prefix New.ReferenceOTU --enable_rev_strand_match

Stdout:

Stderr:

Pick representative set for subsampled failures command
pick_rep_set.py -i otus//step2_otus//subsampled_failures_otus.txt -o otus//step2_otus//step2_rep_set.fna -f otus//step2_otus//subsampled_failures.fasta

Stdout:

Stderr:

Pick reference OTUs using de novo rep set command
pick_otus.py -i otus//step1_otus/failures.fasta -o otus//step3_otus/ -r otus//step2_otus//step2_rep_set.fna -m uclust_ref --enable_rev_strand_match --suppress_new_clusters

Stdout:

Stderr:

Create fasta file of step3 failures command
filter_fasta.py -f otus//step1_otus/failures.fasta -s otus//step3_otus//failures_failures.txt -o otus//step3_otus//failures_failures.fasta

Stdout:

Stderr:

Pick de novo OTUs on step3 failures command
pick_otus.py -i otus//step3_otus//failures_failures.fasta -o otus//step4_otus/ -m uclust --denovo_otu_id_prefix New.CleanUp.ReferenceOTU --enable_rev_strand_match

Stdout:

Stderr:

Merge OTU maps command
cat otus//step1_otus/seqs_otus.txt otus//step3_otus//failures_otus.txt otus//step4_otus//failures_failures_otus.txt > otus//final_otu_map.txt

Stdout:

Stderr:

Pick representative set for subsampled failures command
pick_rep_set.py -i otus//step4_otus//failures_failures_otus.txt -o otus//step4_otus//step4_rep_set.fna -f otus//step3_otus//failures_failures.fasta

Stdout:

Stderr:

Filter singletons from the otu map using API
python -c "import qiime; qiime.filter.filter_otus_from_otu_map('/Users/alaricprins/ITS/otus/final_otu_map.txt', '/Users/alaricprins/ITS/otus/final_otu_map_mc2.txt', '2')"

Write non-singleton otus representative sequences from step1 to the final rep set file: otus//rep_set.fna

Copy the full input refseqs file to the new refseq file
cp its_12_11_otus/rep_set/97_otus.fasta otus//new_refseqs.fna

Write non-singleton otus representative sequences from step 2 and step 4 to the final representative set and the new reference set (otus//rep_set.fna and otus//new_refseqs.fna respectively)

Executing commands.

Make the otu table command
make_otu_table.py -i otus//final_otu_map_mc2.txt -o otus//otu_table_mc2.biom

Stdout:

Stderr:

Executing commands.

Assign taxonomy command
assign_taxonomy.py -o otus//blast_assigned_taxonomy -i otus//rep_set.fna --reference_seqs_fp its_12_11_otus/rep_set/97_otus.fasta --id_to_taxonomy_fp its_12_11_otus/taxonomy/97_otu_taxonomy.txt --assignment_method blast

Stdout:

Stderr:

Executing commands.

Add taxa to OTU table command
biom add-metadata -i otus//otu_table_mc2.biom --observation-metadata-fp otus//blast_assigned_taxonomy/rep_set_tax_assignments.txt -o otus//otu_table_mc2_w_tax.biom --sc-separated taxonomy --observation-header OTUID,taxonomy

Stdout:

Stderr:

qiime_config values:

pick_otus_reference_seqs_fp
/Users/alaricprins/miniconda3/envs/qiime1/lib/python2.7/site-packages/qiime_default_reference/gg_13_8_otus/rep_set/97_otus.fasta
sc_queue all.q
pynast_template_alignment_fp
/Users/alaricprins/miniconda3/envs/qiime1/lib/python2.7/site-packages/qiime_default_reference/gg_13_8_otus/rep_set_aligned/85_otus.pynast.fasta
cluster_jobs_fp start_parallel_jobs.py
assign_taxonomy_reference_seqs_fp
/Users/alaricprins/miniconda3/envs/qiime1/lib/python2.7/site-packages/qiime_default_reference/gg_13_8_otus/rep_set/97_otus.fasta
torque_queue friendlyq
jobs_to_start 1
denoiser_min_per_core 50
assign_taxonomy_id_to_taxonomy_fp
/Users/alaricprins/miniconda3/envs/qiime1/lib/python2.7/site-packages/qiime_default_reference/gg_13_8_otus/taxonomy/97_otu_taxonomy.txt
temp_dir /var/folders/fp/n478ts1n2ps67vp0g30046tr0000gn/T/
blastall_fp blastall
seconds_to_sleep 1

parameter file values:
beta_diversity:metrics bray_curtis
alpha_diversity:metrics observed_otus,chao1
parallel:jobs_to_start 1

Input file md5 sums:
otus/otu_table_mc2_w_tax.biom: 1cebd45b8cf5723e45dd8ef8b1f8de47
map_ITS.txt: 1a75c6b910018029f133843f30a5ae37

Executing commands.

Generate BIOM table summary command
biom summarize-table -i otus/otu_table_mc2_w_tax.biom -o cdout//biom_table_summary.txt

Stdout:

Stderr:

Filter low sequence count samples from table (minimum sequence count: 160) command

filter_samples_from_otu_table.py -i otus/otu_table_mc2_w_tax.biom -o cdout//table_mc160.biom -n 160

Stdout:

Stderr:

Rarify the OTU table to 160 sequences/sample command
single_rarefaction.py -i cdout//table_mc160.biom -o cdout//table_even160.biom -d 160

Stdout:

Stderr:

Executing commands.

Beta Diversity (bray_curtis) command
beta_diversity.py -i cdout//table_even160.biom -o cdout//bdiv_even160/ --metrics bray_curtis

Stdout:

Stderr:
/Users/alaricprins/miniconda3/envs/qiime1/lib/python2.7/site-packages/numpy/core/fromnumeric.py:2645: VisibleDeprecationWarning: `rank` is deprecated; use the `ndim` attribute or function instead. To find the rank of a matrix see `numpy.linalg.matrix_rank`.
  VisibleDeprecationWarning)

Rename distance matrix (bray_curtis) command
mv cdout//bdiv_even160//bray_curtis_table_even160.txt cdout//bdiv_even160//bray_curtis_dm.txt

Stdout:

Stderr:

Principal coordinates (bray_curtis) command
principal_coordinates.py -i cdout//bdiv_even160//bray_curtis_dm.txt -o cdout//bdiv_even160//bray_curtis_pc.txt

Stdout:

Stderr:

Make emperor plots, bray_curtis) command
make_emperor.py -i cdout//bdiv_even160//bray_curtis_pc.txt -o cdout//bdiv_even160//bray_curtis_emperor_pcoa_plot/ -m map_ITS.txt

Stdout:

Stderr:

Executing commands.

Alpha rarefaction command
multiple_rarefactions.py -i cdout//table_mc160.biom -m 10 -x 160 -s 15 -o cdout//arare_max160//rarefaction/

Stdout:

Stderr:

Alpha diversity on rarefied OTU tables command
alpha_diversity.py -i cdout//arare_max160//rarefaction/ -o cdout//arare_max160//alpha_div/ --metrics observed_otus,chao1

Stdout:

Stderr:

Collate alpha command
collate_alpha.py -i cdout//arare_max160//alpha_div/ -o cdout//arare_max160//alpha_div_collated/

Stdout:

Stderr:

Removing intermediate files command
rm -r cdout//arare_max160//rarefaction/ cdout//arare_max160//alpha_div/

Stdout:

Stderr:

Rarefaction plot: All metrics command
make_rarefaction_plots.py -i cdout//arare_max160//alpha_div_collated/ -m map_ITS.txt -o cdout//arare_max160//alpha_rarefaction_plots/

Stdout:

Stderr:
/Users/alaricprins/miniconda3/envs/qiime1/lib/python2.7/site-packages/matplotlib/collections.py:590: FutureWarning: elementwise comparison failed; returning scalar instead, but in the future will perform elementwise comparison
  if self._edgecolors == str('face'):

Skipping compare_alpha_diversity.py as no categories were provided.

Executing commands.

Sort OTU Table command
sort_otu_table.py -i cdout//table_mc160.biom -o cdout//taxa_plots/table_mc160_sorted.biom

Stdout:

Stderr:

Summarize Taxonomy command
summarize_taxa.py -i cdout//taxa_plots/table_mc160_sorted.biom -o cdout//taxa_plots/

Stdout:

Stderr:

Plot Taxonomy Summary command
plot_taxa_summary.py -i cdout//taxa_plots/table_mc160_sorted_L2.txt,cdout//taxa_plots/table_mc160_sorted_L3.txt,cdout//taxa_plots/table_mc160_sorted_L4.txt,cdout//taxa_plots/table_mc160_sorted_L5.txt,cdout//taxa_plots/table_mc160_sorted_L6.txt -o cdout//taxa_plots//taxa_summary_plots/

Stdout:

Stderr:
/Users/alaricprins/miniconda3/envs/qiime1/lib/python2.7/site-packages/matplotlib/collections.py:590: FutureWarning: elementwise comparison failed; returning scalar instead, but in the future will perform elementwise comparison
  if self._edgecolors == str('face'):

Executing commands.

Compress the filtered BIOM table command
gzip cdout//table_mc160.biom

Stdout:

Stderr:

Compress the rarefied BIOM table command
gzip cdout//table_even160.biom

Stdout:

Stderr:

Logging stopped at 15:01:45 on 19 Aug 2017
